# The transfer of 98% of the genome of *Aegilops mutica* into wheat (*Triticum aestivum*)

**DOI:** 10.1007/s00122-026-05173-1

**Published:** 2026-02-09

**Authors:** Julie King, Surbhi Grewal, Caiyun Yang, Duncan Scholefield, Stephen Ashling, Manel Othmeni, Katie Hawkins, Ian P. King

**Affiliations:** Nottingham Wheat Research Centre, School of Biosciences, University of Nottingham, Loughborough, LE12 5RD UK

## Abstract

**Key message:**

New wheat-*Ae. mutica* introgression lines will deliver new genetic variation for hexaploid wheat breeding and provide new information on the distribution of homoeologous recombination between wheat and *Ae. mutica*.

**Abstract:**

*Aegilops mutica* Boiss. (2n = 2 × = 14, TT) is a wild relative of wheat that has been underutilised as a source of genetic variation for hexaploid wheat *Triticum aestivum* L. (2n = 6 × = 42; AABBDD), despite its potential to harbour important genetic diversity for a wide range of agronomically valuable traits. This species has been extensively exploited by the Wheat Research Centre (WRC) at the University of Nottingham to create a diverse resource of wheat-*Ae. mutica* introgression lines. In this study, we present the most comprehensive transfer of the *Ae. mutica* genome into wheat to date, with 98% of the genome now present in wheat through the development of new wheat–*Ae. mutica* introgression lines. These 68 new lines, comprising 57 unique *Ae. mutica* introgressions, have been characterised using kompetitive allele-specific PCR (KASP) genotyping, multi-colour genomic in situ hybridisation and low coverage whole-genome sequencing. This thorough characterisation has revealed the distribution of homoeologous recombination sites between wheat and *Ae. mutica* chromosomes, uncovering recombination “hotspots” and novel introgressed segments that were previously undetectable using conventional genotyping methods. This resource significantly expands the genetic diversity available for wheat improvement and offers a powerful platform for linking traits to specific genotypes. The creation and characterisation of this near-complete set of *Ae. mutica* introgressions will be invaluable for wheat researchers and breeders worldwide.

**Supplementary Information:**

The online version contains supplementary material available at 10.1007/s00122-026-05173-1.

## Introduction

The wild relatives of wheat provide a vast reservoir of genetic variation for virtually all traits of agronomic importance. Transfer of the genetic variation from the wild relatives in the form of chromosome segments or introgressions is not a new science, but dates back many decades. Indeed, a paper by McFadden and Sears of 1047, outlines a proposal by Carlton (1901) to exploit variation from wild relatives. Genetic variation transferred from wild relatives has had a major impact on wheat improvement (King et al. 2024) for a range of traits of increasing importance for food security due to climate change and global warming. Traits of importance include resistance to abiotic stresses such as increased heat (Giovenali et al. 2024; Molero et al. 2023) and drought tolerance (Lu et al. 2022) and resistance to pathogens such as stem rust race Ug99 (Mago et al. 2009), and diseases such as eyespot (Doussinault et al., 1983), powdery mildew (Liu et al. 2017), etc. Furthermore, the wild relatives have the potential to provide genetic variation to overcome new and emerging threats to wheat production particularly where little or none is present in the gene pool of wheat, e.g., an introgression from *Aegilops ventricosa* is currently providing the best source of resistance to wheat blast (Cruz et al. 2016).

The development of new technologies has provided the means of increasing the scale of the transfer so that it is now possible to introgress large numbers of chromosome segments from a wild relative into wheat, identify and characterise the introgressions and very importantly, track the introgressions throughout each generation in breeding programmes. The WRC at Nottingham has developed a large number of chromosome-specific KASP markers (Grewal et al. 2020a); markers designed to be polymorphic between wheat and different wild relatives and able to determine the zygosity of the introgressions in wheat. The density of markers has been increased for several of the species under study at Nottingham, including *Ae. mutica* (the target being the generation of one KASP marker for every 50Mbp between wheat and each wild relative) (Grewal et al. 2022).

*Ae. mutica* (2n = 2x = 14, TT) has been widely exploited by the WRC to create a diverse resource of wheat-*Ae. mutica* introgression lines (King et al. 2017, 2019). The classification of *Ae. mutica* has been much debated with some arguing that it is closely related to *Aegilops speltoides* and should therefore be included in the Sitopsis, and others arguing that it sits by itself as the only member of the genus Amblyopyrum. Recently, published work confirms that *Ae. mutica* is indeed closely related to *Ae. speltoides* (Bernhardt et al., 2020; Li et al. 2022).

In the past, *Ae. mutica* has been underutilised as a source of genetic variation for wheat improvement. However, the species has enormous potential, e.g. the first fifteen introgression lines generated at Nottingham were shown to carry resistance to stripe, leaf and stem rust (Fellers et al. 2020). In addition, initial analyses have shown they also carry variation for a range of other traits including heat tolerance, powdery mildew resistance, wheat blast resistance and high photosynthetic potential (unpublished data). Furthermore, *Ae. mutica* also carries a gene(s) that promotes recombination between homoeologous chromosomes even in the presence of the *Ph1* pairing control locus located on the long arm of chromosome 5B of wheat (Dover and Riley 1972a and b). These promotors facilitate recombination between homoeologous chromosomes of wheat and *Ae. mutica*, hence leading to the generation of introgressions which are recovered in the offspring (King et al. 2017).

KASP markers developed at Nottingham were initially used to identify and characterise 67 wheat-*Ae. mutica* doubled haploid lines (Grewal et al. 2022). These markers are well spread across the genome of wheat, with an average distance between markers of 26 Mb and only seven instances where the distance between two markers exceeded 70 Mb. The markers can distinguish between heterozygous and homozygous introgressions, unless the introgression is present as an addition in which case the KASP markers detect the introgression as heterozygous due to the presence of all homoeologous wheat chromosomes of the same linkage group in wheat (Grewal et al. 2020a). A few of these doubled haploid lines were further characterised by whole-genome sequencing to delimit introgression regions using sequence read coverage analysis (Coombes et al. 2023).

In this paper, we describe the characterisation, using KASP analysis, of an additional 68 new *Ae. mutica* introgression lines that when combined with the original 67 lines (King et al. 2017, 2019) represent 98% of the total genome of this species. Furthermore, to determine the efficacy of the KASP marker panel and to obtain additional information, the existing and new introgressions were all analysed with low coverage whole-genome sequencing or “skim-sequencing” and screened with multi-colour genomic in *situ* hybridisation (multi-colour GISH). This combined genotyping approach provided new information on the distribution of homoeologous recombination sites that occurred between wheat and *Ae. mutica* chromosomes and uncovered smaller introgressions that were not detected solely by KASP analysis.

## Material and methods

*Plant material*: Introgression lines were generated as described in King et al. (2017) by crossing three accessions of *Ae. mutica* (2,130,004, 2,130,008 and 2,130,012 obtained from the Germplasm Resource Unit (GRU) at the John Innes Centre (JIC)) as the pollen parent with hexaploid wheat cv. Paragon (GRU, JIC) as the female parent. (The majority of the initial crosses were made with accessions 2,130,004 and 2,130,012 and the fewest with accession 2,130,008 due to matching of male and female plants in the glasshouse during the original crossing programme.) F_1_ hybrids were grown to maturity and pollinated with Paragon to generate BC_1_ plants, which were then recurrently backcrossed. Genotyping analysis of the early backcross lines was carried out using the 36 K Axiom® Wild-Relative Genotyping Array (King et al. 2017) to establish which plants carried introgressions from *Ae. mutica*. The advanced backcross generations were screened with a set of chromosome-specific KASP markers (Grewal et al. 2022) to identify plants carrying unique introgressions. Introgressions present in different BC_1_ plants were generated by independent recombination events during gamete formation in the F_1_ hybrids and therefore introgressions generated from different BC_1_ plants were considered to be unique even when of a similar size. Plants of interest were self-fertilised and screened with KASP markers and multi-colour GISH after each round of self-fertilisation until all introgressions were homozygous.

All plants were grown in a glasshouse in 2-L pots containing John Innes No. 2 soil and maintained at 18–25 °C under 16-h light and 8-h dark conditions.

*Genomic DNA extraction*: Leaf material (1.5-inch leaf segments cut into pieces) was collected from young 2-week-old plants into a deep-well plate, freeze-dried and ground to a fine powder in a TissueLyser II (Qiagen) at a frequency of 25 Hz for 4 min. Extraction of genomic DNA for KASP genotyping and skim-sequencing was carried out as described in Grewal et al. (2022) with an additional purification step with phenol/chloroform at the end of the protocol for the extraction of DNA used for the generation of probes for multi-colour GISH.

*KASP marker analysis*: The genotyping protocol was carried out as described in Grewal et al. (2020b). Briefly, an automated PIPETMAX 268 (Gilson) was used to set up the genotyping reactions and performed in a ProFlex PCR system (Applied Biosystems by Life Technology) in a final volume of 5 μl with 1 ng genomic DNA, 2.5 μl KASP reaction mix, 0.068 μl primer mix and 2.43 μl nuclease-free water. Polymerase chain reaction (PCR) conditions were set as 15 min at 94 °C; 10 touchdown cycles of 10 s at 94 °C, 1 min at 65–57 °C (dropping 0.8 °C per cycle); and 35 cycles of 15 s at 94 °C, 1 min at 57 °C. Fluorescence detection of the reactions was performed using a QuantStudio 5 (Applied Biosystems) and the data analysed using the QuantStudio™ Design and Analysis Software V1.5.0 (Applied Biosystems).

*Multi-colour GISH*: Preparation of the root-tip metaphase chromosome spreads, the protocol for multi-colour GISH and the image capture was as described in Grewal et al. (2020b). Briefly, genomic DNA was extracted from *Ae. mutica*, *T. urartu* (for detection of the A-genome), *Ae. speltoides* Tausch (for detection of the B-genome) and *Aegilops tauschii* Coss. (for detection of the D-genome) as described above and labelled by nick translation with ChromaTide™ Alexa Fluor™ 546–14-dUTP (Invitrogen; C11401; coloured yellow), ChromaTide™ Alexa Fluor™ 488–5-dUTP (Invitrogen; C11397; coloured green), DEAC-dUTP (Jena Bioscience; NU-803-DEAC; coloured blueish purple) and ChromaTide™ AlexaFluor™ 594–5-dUTP (Invitrogen; C11400; coloured red), respectively. Slides were probed using 150 ng of *T. urartu*, 150 ng of *Ae. speltoides*, 300 ng of *Ae. tauschii* and 50 ng of *Ae. mutica*-labelled genomic DNAs, in the ration 3:3:6:1. Slides were counterstained using 4’,6-diamidino-2phenylindole,dihydrochloride (DAPI) and analysed using a fully automated Zeiss Axio ImagerZ2 upright epifluorescence microscope (Carl Zeiss Ltd). Image capture was performed using a Coolcube 1-m CCD camera (Metasystems, Altlussheim, Germany), and image analysis was carried out using Metafer4 (automated metaphase image capture) and ISIS (image processing) software (Metasystems).

*Skim-sequencing and introgression mapping*: The library preparation and sequencing were carried out by Novogene, UK, Ltd. Genomic DNA was randomly sheared into shorter fragments. The obtained fragments were then end-repaired, A-tailed, and further ligated with Illumina adapters. The resulting fragments with adapters were size selected, PCR amplified before proceeding for purification. The libraries were quantified through Qubit and qPCR, and size distribution detected with a fragment analyser. Quantified libraries were pooled and sequenced on Illumina NovaSeq 6000 S4 flowcells to produce 150 bp paired-end reads with an average coverage of 0.05 × per library.

A combined wheat–alien reference mapping pipeline was implemented to identify *Ae. mutica* introgressions in wheat lines, building on workflows described by Coombes et al. (2023) and Adhikari et al. (2022). Paired-end Illumina reads from each introgression line and two wheat parents (e.g. Chinese Spring and Paragon) were adapter- and quality-trimmed using Trimmomatic (Bolger et al. 2014). The trimmed reads were aligned to a concatenated reference genome containing the wheat RefSeq v2.1 assembly (Zhu et al. 2021) and the recently assembled *Ae. mutica* genome (Grewal et al. 2025) using HISAT2 (Kim et al. 2015) with the parameters—no-spliced-alignment—no-unal.

Properly paired alignments were extracted using SAM flag filtering, retaining uniquely mapped reads with mapping quality ≥ 10. Duplicate reads were removed using Picard (Broad Institute), and the resulting BAM files were indexed using SAMtools (Li et al. 2009). To quantify coverage, 1 Mb non-overlapping genomic windows were generated using BEDTools (Quinlan and Hall 2010), and read counts in each window were computed using hts_nim_tools.

Coverage deviation for each window was calculated using a custom Python script (coverage_deviation_combined.py). For wheat chromosomes, the coverage of each line was normalised against the genome-wide medians of the two wheat parents, and deviation was computed relative to the parent with the most similar baseline. For *Ae. mutica* chromosomes, coverage was normalised against a dynamic baseline derived from the upper decile of non-zero bins within the line itself. This approach adjusts for variable sequencing depth and enables comparability across samples.

**Fig. 1:**
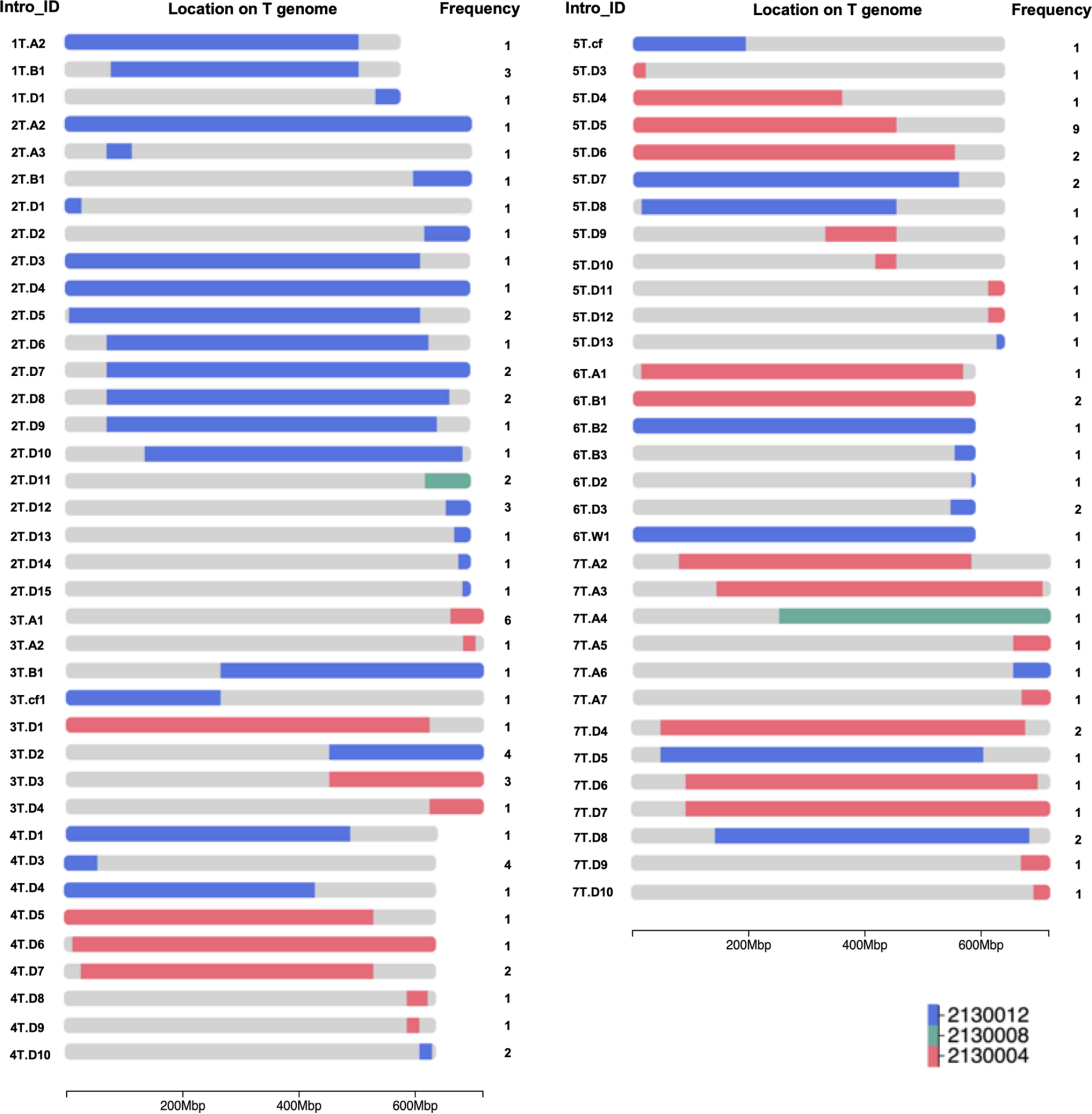
Characterised introgressions from *Ae. mutica* in the 68 new WRC_Mut lines. The name (corresponding to Table 1), size, location and frequency of each of the new *Ae. mutica* introgression into the wheat A-, B- and D-genomes for each wheat homoeologous group. Introgressions from *Ae. mutica* accession 2130004 are shown in red, accession 2130008 in green and accession 2130012 in blue

Deviation values close to 1 indicate typical chromosomal representation in both wheat and *Ae. mutica* genomes (Fig. 1S). On wheat chromosomes, values below 1 suggest a loss of wheat chromatin, approximately 0.5 for heterozygous deletions and near 0 for homozygous deletions. However, such drops are only considered indicative of *Ae. mutica* introgression if accompanied by a corresponding increase in coverage on a homoeologous *Ae. mutica* chromosome (in most cases). Homoeology was inferred based on shared chromosome group numbers (e.g. 5A and 5 T), assuming collinearity. On *Ae. mutica* chromosomes, values are normally near 0 in the absence of introgression, while deviation values greater than 0.2, approaching 2, indicate the presence of *Ae. mutica* chromatin in the wheat background (Fig. 1S).

Coverage deviation profiles were visualised in R (ggplot2) (Fig. 1S). Wheat regions with sustained drops in coverage (< 0.1 across ≥ 6 consecutive windows) were coloured red only if supported by prior detection of a corresponding increase in *Ae. mutica* coverage (> 0.2 across ≥ 5 bins), indicating a probable introgression.

## Results

### Characterisation of wheat-*Ae. mutica* introgression lines

**Fig. 2:**
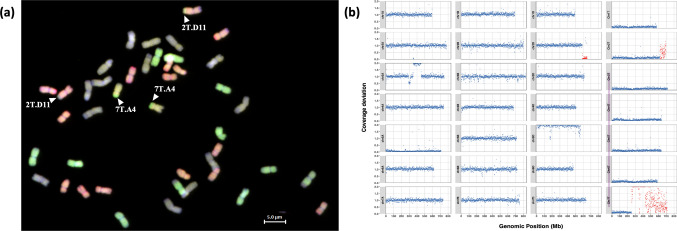
Detection of *Ae. mutica* introgressions in introgression line Mut15 using combined reference mapping. **a** Multi-colour GISH metaphase spread of Mut15 showing chromosomes from the A-genome (green), B-genome (purple), D-genome (red), and *Ae. mutica* T-genome (yellow). Two T-genome introgressions are visible: 2 T.D11, where the 2 T segment has recombined with wheat chromosome 2D, and 7 T.A4, where the 7 T segment appears added to a wheat chromosome from the A subgenome of wheat. **b** Coverage deviation plots from the combined wheat + *Ae. mutica* reference mapping pipeline. Red points indicate regions with abnormal coverage. On the wheat genome, a drop in coverage along 2D indicates loss of wheat chromatin due to the 2 T introgression. The 7 T segment is clearly visible as a gain in coverage on chromosome 7 T of *Ae. mutica*, despite no corresponding drop in wheat group 7 chromosomes, suggesting that the 7 T introgression has undergone a non-homoeologous translocation event. A duplicated region on wheat chromosome 3A (coverage ~ 2) supports the interpretation that the 7 T segment has translocated with this region rather than with 7A

The 68 new wheat-*Ae. mutica* homozygous introgression lines were characterised using KASP genotyping, multi-colour GISH and skim-sequencing to find 57 new unique introgressions from *Ae. mutica* (Figs. 1 and 2S) (whole chromosomes are not included in this total)*.* KASP genotyping was carried out using 310 chromosome-specific markers polymorphic between *Ae. mutica* and wheat (Grewal et al. 2020a; Grewal et al. 2022; Table 1S). Multi-colour GISH characterisation, in tandem with the KASP markers, was able to confirm the lines that carried an additional pair of complete *Ae. mutica* chromosomes. All lines were also skim-sequenced to a resolution of 0.05 × coverage. All three methods of characterisation were found to be useful, with no one method providing all the information required for a full characterisation of the introgression and wheat background in the introgression lines. The introgressions in the new lines were coded according to their size and the wheat genome they had recombined with (Fig. 1; Tables 1 and 2S). The coding system was continued from the codes previously used for the *Ae. mutica* introgressions reported by Grewal et al. (2022). The average and median introgression sizes were ~ 250 Mbp and 76 Mbp, respectively, with the smallest being 1.42 Mbp and the largest being 636 Mbp (not including whole chromosome additions) from Chr 6 T and Chr 7 T of *Ae. mutica*, respectively (Table 3S).

**Table 1:** List of introgressions in the 68 new homozygous introgression lines detected by KASP genotyping, multi-colour GISH and skim-sequencing. Where introgression lines contain more than one introgression, the different introgressions are shown as Intro (introgression) 1, 2, etc, in the table. The maximum number of introgressions present in any one line was four

Line code	Numbers and classification of introgressions in each line (Codes for each introgression as in Figure 1)				Line code	Numbers and classification of introgressions in each line (Codes for each introgression as in Figure 1)			
	Intro 1	Intro 2	Intro 3	Intro 4		Intro 1	Intro 2	Intro 3	Intro 4
WRC21_Mut2	2T.A2				WRC21_Mut39	4T.D7	4T.D9	5T.D5	7T.D7
WRC21_Mut3	2T.D3				WRC21_Mut40	2T.D1			
WRC21_Mut4	7T.D5				WRC21_Mut41	3T.D4	7T.D4		
WRC21_Mut5	2T.D10	6T.D3			WRC21_Mut42	3T.D2			
WRC21_Mut6	2T.D12	3Tcf	5T.cf		WRC21_Mut43	3T.D2			
WRC21_Mut7	1D.D1				WRC21_Mut44	3T.A1	5T.D5		
WRC21_Mut10	2T.B1	4T.D4	4T.D10*	5T.D13	WRC21_Mut45	3T.A1	5T.D5		
WRC21_Mut12	4T.D3				WRC21_Mut46	3T.D1			
WRC21_Mut13	5T.D8	7T.A6			WRC21_Mut47	2T.D5			
WRC21_Mut14	2T.D2				WRC21_Mut48	4T.D1			
WRC21_Mut15	2T.D11	7T.A4			WRC21_Mut49	3T.A1	5T.D3	5T.D10	7T.A7*
WRC21_Mut16	2T.D11				WRC21_Mut50	3T.B1	7T.D8		
WRC21_Mut17	2T.D9				WRC21_Mut51	2T.D5			
WRC21_Mut18	2T.D8				WRC21_Mut52	6T.A1			
WRC21_Mut19	2T.D7				WRC21_Mut53	7T.D4			
WRC21_Mut20	2T.D7				WRC21_Mut54	7T.A2			
WRC21_Mut21	2T.A3	2T.D6	2T.D15		WRC21_Mut55	5T.D4			
WRC21_Mut22	4T.D3				WRC21_Mut56	5T.D5			
WRC21_Mut23	3T.D2				WRC21_Mut57	7T.A5			
WRC21_Mut24	4T.D5	7T.D10*			WRC23_Mut58	5T.D5			
WRC21_Mut25	4T.D6				WRC23_Mut62	2T.D4			
WRC21_Mut26	4T.D3				WRC23_Mut66	4T.D3			
WRC21_Mut27	3T.A1	5T.D5			WRC23_Mut70	1T.A2			
WRC21_Mut28	3T.D2				WRC23_Mut73	1T.B1	4T.D10		
WRC21_Mut29	2T.D12	5T.D7			WRC23_Mut74	3T.D3			
WRC21_Mut30	3T.A1	5T.D5			WRC23_Mut75	4T.D7	5T.D5	7T.D6	
WRC21_Mut31	1T.B1				WRC23_Mut77	4T.D8*	5T.D5	7T.D9	
WRC21_Mut32	1T.B1	6T.B2			WRC23_Mut78	2T.D12	5T.D7		
WRC21_Mut33	6T.W1				WRC23_Mut79	2T.D13	7T.D8		
	add								
WRC21_Mut34	5T.D11	6T.B1			WRC23_Mut80	3T.A2	7T.A3		
WRC21_Mut35	3T.D3				WRC23_Mut82	3T.A1	5T.D9		
WRC21_Mut36	3T.D3	5T.D6			WRC23_Mut84	2T.D8			
WRC21_Mut37	5T.D6				WRC23_Mut85	2T.D14	6T.D3		
WRC21_Mut38	6T.B1				WRC24_Mut86	6T.B3*			

A = A-genome, B = B-genome, D = D-genome, T = *Ae. mutica*, cf = centric fusion, add = addition chromosome.

*Detected by skim-sequencing but not KASP and GISH

Introgression mapping was carried out using a combined reference-based pipeline that simultaneously assessed the presence or absence of wheat and *Ae. mutica* chromatin in each introgression line (Coombes et al. 2023; Adhikari et al. 2022). By mapping skim-sequencing reads to a concatenated reference genome comprising the wheat RefSeq v2.1 assembly (Zhu et al. 2021) and the *Ae. mutica* genome (Grewal et al. 2025), and calculating normalised coverage deviation across 1 Mb windows, both losses of wheat chromatin and gains of *Ae. mutica* segments were precisely identified (Table 2S; Fig. 1S). This dual analysis not only revealed which regions of the wheat genome were missing but also confirmed which *Ae. mutica* chromosomes had been introgressed. Using this method, we confirmed the presence of all introgressions identified by KASP genotyping and GISH and identified new small introgressions (Table 1; Fig. 1).

In the case of Mut15, two homozygous introgressions were observed by multi-colour GISH, one associated with the D-genome and the other with the A-genome (Table 1; Fig. 2a). Sequence coverage deviation profiles confirmed the presence of the 2 T segment, which had clearly recombined with chromosome 2D (Fig. 2b). For the 7 T segment, KASP genotyping indicated a heterozygous state due to the presence of all three group 7 wheat homoeologs (7A, 7B, 7D). Coverage deviation analysis confirmed that all three wheat group 7 chromosomes were intact, with no loss of wheat chromatin observed in this group. However, a distinct increase in coverage was detected on the *Ae. mutica* 7 T chromosome, indicating that the 7 T segment was indeed present in the introgression line. But the lack of a corresponding drop in group 7 wheat chromosomes suggested that the 7 T segment had not replaced its homoeologous counterpart. Instead, the data indicated that the 7 T introgression had undergone a non-homoeologous translocation event with a ~ 100-Mb proximal region of chromosome 3A which showed increased coverage consistent with a duplication (Fig. 2b).

**Fig. 3:**
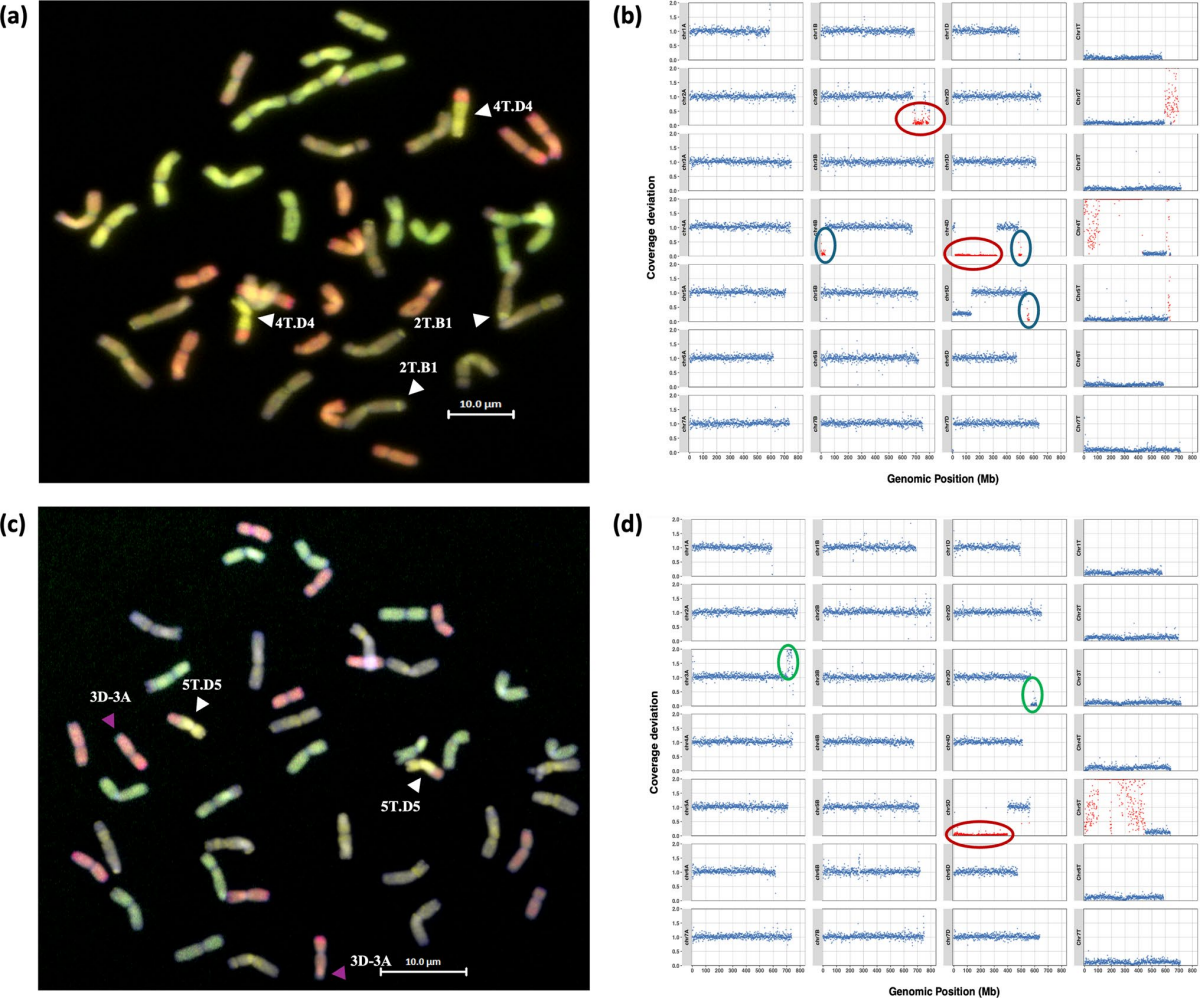
GISH and coverage deviation analysis of homozygous introgression lines Mut10 and Mut58. The A-genome chromosomes are shown in green, the B-genome in purple, the D-genome in red, and the T-genome in yellow in GISH images. In the coverage deviation plots, out of bound values on the y-axis were plotted at 2. **a** GISH of a metaphase spread of Mut10 showing the two large introgressions from chromosomes 2 T and 4 T. **b** Skim-sequencing data showing smaller introgressions previously undetected by KASP or GISH (blue circles). Skim-sequencing identifies a small 5 T introgression into chromosome 5D and a smaller 4 T introgression into chromosome 4DL. A possible coverage drop on the short arm of chromosome 4B may represent a small undetected segment the result of an earlier recombination with 4 T, although this remains speculative. The two large introgressions from 2 and 4 T detected by KASP, GISH, and skim-sequencing are also highlighted (red circles). **c** GISH of a metaphase spread of Mut58 showing a 5 T introgression into 5D and an A–D recombination event (yellow arrows). **d** Coverage deviation in Mut58 confirming the 5 T introgression (red circle) and revealing an inter-genomic translocation between chromosomes 3A and 3D (green circles), where a duplicated region from the end of 3AL has replaced a deleted region on 3DL. This event was not detected by KASP genotyping and only partially resolved by GISH

Skim-sequencing identified the presence of five small *Ae. mutica* segments that were not detected using the KASP markers and GISH (Table 1). For example in Mut10, a small 4 T segment of 17Mbp, visible as a clear gain in coverage on the long arm of chromosome 4 T (Fig. 3b), appears to have recombined into the wheat genome at the distal end of chromosome 4DL but was undetectable by GISH (Fig. 3a). In addition, the larger 4 T.D4 introgression begins at the start of chromosome 4TS. However, the distal end of 4DS appears intact. One possible explanation is that the 4 T segment initially recombined into the short arm of chromosome 4B, followed by a secondary recombination event with 4D, effectively splitting the introgression across two wheat group 4 chromosomes. However, due to the small size of the segment on 4BS and lack of detectable signal via GISH, this remains speculative and is therefore noted as one large introgression on 4D (Table 1). A small introgression of size 13 Mbp from chromosome 5 T into the distal end chromosome 5DL was also detected by skim-sequencing (Fig. 3b). All the additional introgressions detected with the skim-sequencing occured within regions between two KASP markers, resulting in the non-detection of these segments during initial KASP genotyping (Table 1S). Based on these results, KASP markers have now been designed that are able to detect and track these smaller segments (Table 4S).

GISH analysis of the metaphase spreads of the homozygous introgression lines also showed chromosomes derived from homoeologous recombination between wheat chromosomes. In total, skim-sequencing was able to identify 50 inter-genomic recombination events in the new homozygous lines and also which wheat chromosomes had recombined and the size of the translocations (Table 5S). For example, in Mut58, GISH showed a small A-genome segment at the end of the long arm of a pair of D-genome chromosomes (Fig. 3c) and skim-sequencing confirmed this as homoeologous recombination between wheat chromosomes 3AL and 3DL (Fig. 3d) where 3DL chromatin had been replaced by a duplicated segment from 3AL.

### Transfer of *Ae. mutica* genome into wheat

In a previous study, we report the generation of 67 wheat-*Ae. mutica* introgression lines (King et al. 2019). Grewal et al. (2022) used high-density KASP markers to find that only 51 of the 67 lines carried *Ae. mutica* introgressions with a total of 14 unique introgressions. In this work, the skim-sequencing analysis was extended to include these 51 previously generated introgression lines confirming these 14 introgressions and also identifying one new introgression in DH-203 (5 T.D12) (Table 2S).

The 57 new introgressions described and characterised here equate to 98% of the *Ae. mutica* genome having been transferred into wheat. However, a total of 98.1% of the *Ae. mutica* genome has been transferred into wheat when these new introgressions are combined with the 14 introgressions reported previously (Grewal et al. 2022; Fig. 3S; Table 2S). When all introgressions are considered, chromosomes 2 T, 3 T, 4 T, 6 T and 7 T have been transferred in their entirety while the missing 2% comprises one gap of approximately 37 Mbp on chromosome 1 T and 1 gap of 51 Mbp on chromosome 5 T although a whole 1 T chromosome has also been transferred into wheat as part of the previously available set of introgression lines (line DH-161).

The Nottingham crossing programme has used three different accessions of *Ae. mutica*. The new introgressions transfer 70% of accession 2,130,004 into wheat, 12% of accession 2,130,008 and 85% of accession 2,130,012 made up of 28 unique introgressions from 2,130,004, 2 from 2,130,008 and 27 from 2,130,012 (Table 2). When the total introgressions generated are considered, the transfer of accession 2,130,012 is increased to 86% while the transfer of accessions 2,130,004 and 2,130,008 remain as 70% and 12%, with the total numbers of unique segments from 2,130,004, 2,130,008 and 2,130,012 being 30, 2 and 39 respectively. The highest number of introgressions were generated from chromosomes 2 T and 7 T (17) *Ae. mutica* and the fewest from chromosome 1 T (3) (Table 2 and 3S). The lines described are homozygous for all introgressions. At the Nottingham WRC, further unique introgressions have been generated and are presently being self-fertilised to make them homozygous prior to distribution.

**Table 2:** Number of homozygous introgressions generated from each chromosome of the three accessions of *Ae. mutica *(2130004, 2130008 and 2130012). The numbers include both the new introgressions plus those previously reported (shown in brackets). The number of recombination hotspots and the number of further recombination sites are also given for each *Ae. mutica* chromosome

Chromosome	Number of introgressions (previously published)	Total number of introgressions	Recombination hot spots	Further recombination sites
1T	0 (0)^4^ 0 (0)^8^ 3 (0)^12^	3	1	0
2T	0 (0)^4^ 1 (0)^8^ 13 (3)^12^	17	1	12
3T	5 (0)^4^ 0 (0)^8^ 2 (0)^12^	7	1	5
4T	5 (0)^4^ 0 (0)^8^ 2 (4)^12^	11	0	7
5T	8 (0)^4^ 0 (0)^8^ 2 (2)^12^	12	1	9
6T	1 (0)^4^ 0 (0)^8^ 2 (1)^12^	4	0	3
7T	9 (2)^4^ 1 (0)^8^ 3 (2)^12^	17	4	15
Total	28 (2)^4^ 2 (0)^8^ 27 (12)^12^	71	8	51

^4^—Accession 2130004; ^8^—Accession 2130008; ^12^—Accession 2130012

### Recombination between *Ae. mutica* and bread wheat chromosomes

In the previous study, we reported that recombination between the chromosomes of *Ae. mutica* and the wheat B and D-genomes in the set of doubled haploid lines was approximately equal but that we had seen no recombination between *Ae. mutica* and the A-genome of wheat (King et al. 2019). However, this work showed that 18% of introgressions had recombined with the A-genome, 7% with the B-genome and 75% with the D-genome (Table 2S).

**Fig. 4:**
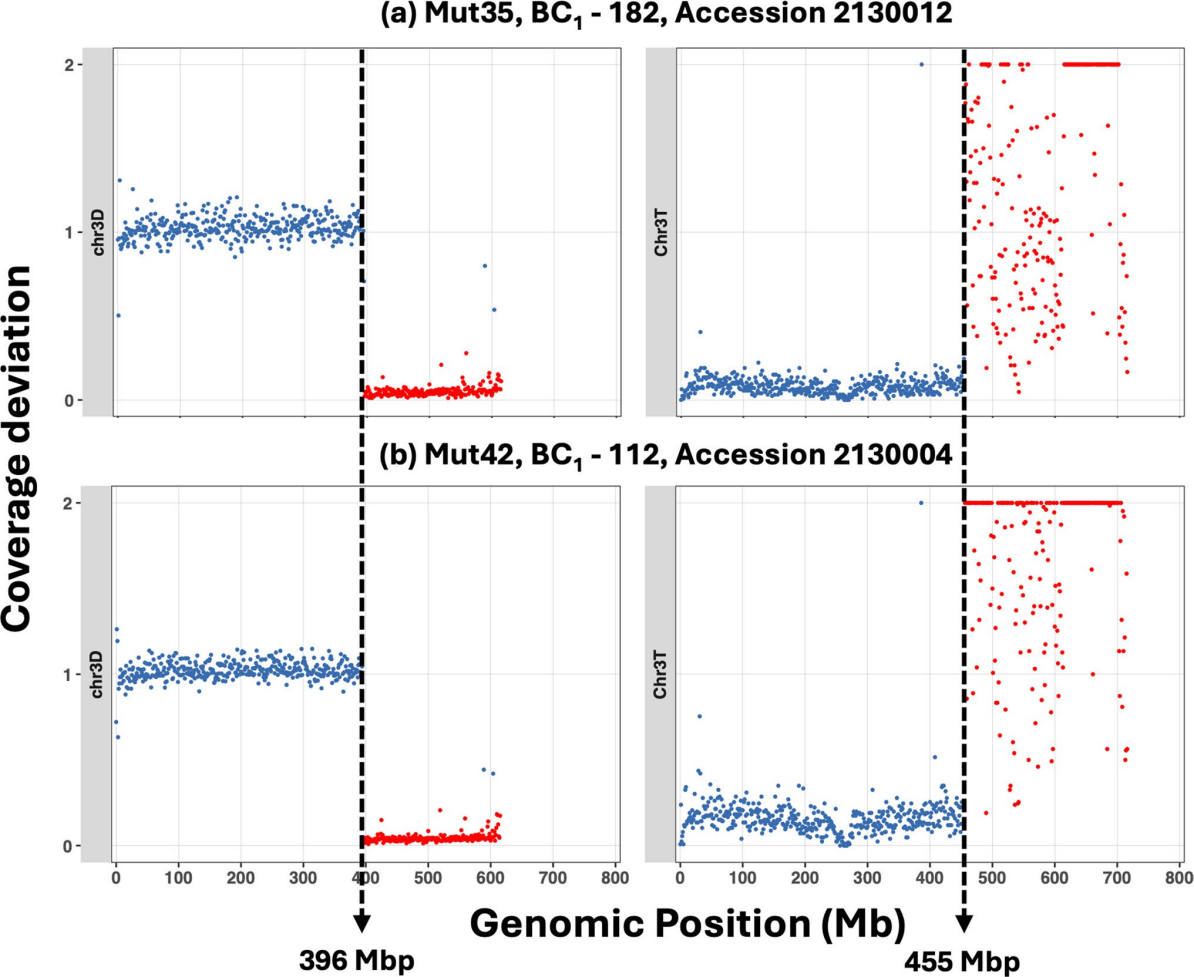
Skim-sequencing showing a hotspot for recombination between chromosomes 3D of wheat and 3 T of *Ae. mutica*. **a** Mut35 and **b** Mut42 were generated from two different BC_1_ plants produced from different *Ae. mutica* accessions. Out of bound values on the y-axis were plotted at 2

The new homozygous introgression lines reported in this paper were generated from 17 individual BC_1_ plants. Introgressions present in the original 17 BC_1_ plants were generated via recombination in the F_1_ gametes and therefore generated by independent recombination events. Skim-sequencing revealed that some sites of recombination occurred in very similar positions (as far as it was possible to identify from the resolution of skim-sequencing) in most chromosomes, of wheat and those of *Ae. mutica* (Fig. 4). A potential 8 hotspots were found spread between chromosomes 1 T, 2 T, 3 T, 5 T and 7 T (Table 2 and 6S). Where hotspots of recombination were detected in the wheat genome, corresponding hotspots occurred in the mutica genome (Fig. 4). However, two hotspots were found in *Ae. mutica* (1 T and 7Tiv) where *Ae. mutica* had recombined with different genomes of wheat. Thus, it is probable that the wheat junctions given do represent similar regions of the different wheat chromosomes. The hotspots of recombination were found when comparing not only *Ae. mutica* introgression lines developed from different BC_1_ plants, but also from different *Ae. mutica* accessions. For example, skim-sequencing revealed a recombination hotspot between Mut35 and Mut42, lines generated from two different BC_1_ plants produced from different accessions (Fig. 4a and b).

**Fig. 5:**
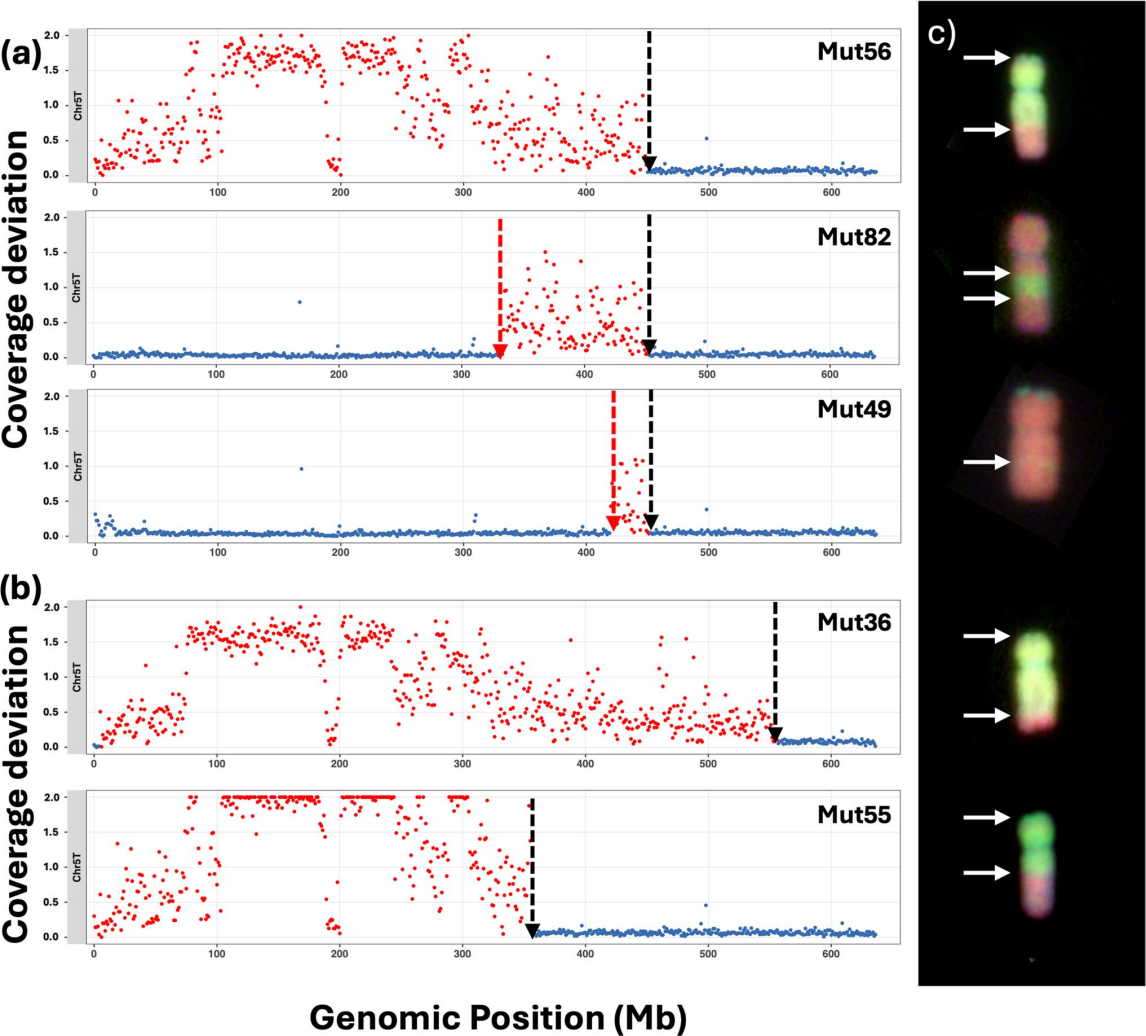
Introgressions from *Ae. mutica* Chr5T **a** showing further recombination between wheat and an *Ae. mutica* introgression in later backcrossed lines to result in smaller introgressions (red arrows), **a** and **b** showing different sites of interstitial recombination (red and black arrows in **a** and black arrows in **b**) and **c** introgressions represented by bright green/yellow regions in white arrows in GISH images of introgression lines with corresponding skim-sequencing plots of Chr5T on the left. Out of bound values on the y-axis of the skim-sequencing plots were plotted at 2

Recombination, however, was not limited to the hotspots, but was also observed across much of the genome (Fig. 5) in addition to the expected telomeric regions. In introgressions from all T chromosomes, except 1 T, additional homoeologous recombination between heterozygous *Ae. mutica* introgressions and wheat chromosomes occurred during the formation of gametes in later generations resulting in the formation of further new introgressions (Table 2). For example, Mut56, Mut82 and Mut49 (Figs. 5a and c) were generated from the same BC_1_ plant and share a pedigree up to the BC_3_ generation. The 5 T introgression in Mut56 is the largest (450 Mbp) and therefore was probably the original introgression. During backcrossing, Mut82 and Mut49 were generated by further recombination events reducing the size of the introgression to 116 Mbp and 30 Mbp, respectively. The majority of homoeologous recombination between the chromosomes of *Ae. mutica* and wheat were located distally towards the telomeres (Table 2S). However, recombination was also found to have occurred in the interstitial regions in some chomosomes, with Fig. 5b and c showing these events in chromosome 5 T.

## Discussion

The 119 *Ae. mutica* homozygous introgression lines developed at the Nottingham WRC (68 new homozygous lines + 51 reported previously) represent a unique source of new genetic variation for utilisation in wheat improvement. *Ae. mutica* is an obligate outbreeder and therefore as a cross-pollinating species is expected to carry high levels of genetic variation (Dvorak et al. 1998). This was confirmed in studies by Alsaleh et al. (2022) and Sasanuma et al. (2002) although Hegde et al. (2002) found less genetic diversity than expected. The original crossing programme at the WRC utilised several individuals from each of the three accessions of *Ae. mutica* and thus even similar sized introgressions from the same original accession might carry different alleles. An introgression was thus considered unique if its pedigree showed it had been generated from a different BC_1_ plant to any other similar sized introgressions, and thus, the 119 homozygous lines carry a total of 71 unique introgressions (14 from the 51 lines reported previously and 57 in the new homozygous lines reported here) (Table 1; Figs. 1 and Fig. 1S and Table 2S).

In our hands, the smallest introgressions detectable with GISH were ~ 15Mbp. The resolution of the KASP markers used to detect introgressions was directly related to the number we had developed and their spread across the wheat genome (Table 1S). Thus, small introgressions that occurred between two flanking KASP markers and were less than 15Mbp in size would not be detected by either the KASP markers or the GISH. Skim-sequencing provided the most complete means of characterisation of each of the lines (Table 2S), although the KASP genotyping, and multi-colour GISH provided additional information for a number of the lines, e.g. Mut15 (Fig. 2). Skim-sequencing was able to detect five introgressions that were not identified using the KASP genotyping and multi-colour GISH (Figs. 3a and b). To enable the future exploitation of these small introgressions in breeding programmes, KASP markers have been designed using the information from the skim-sequencing (Tables 4S).

Multi-colour GISH was used to count the number of wheat chromosomes present in each introgression line and more specifically the number of chromosomes from each wheat genome. Where possible, lines were selected with 42 chromosomes, e.g., a line carrying a homozygous introgression recombined with the D-genome would be expected to carry 14 A chromosomes, 14 B chromosomes, 12 D chromosomes and 2 D-T introgression chromosomes. Skim-sequencing confirmed the genomic constitution of the wheat genome in the introgression lines, including the presence of any deletions and/or duplications. Skim-sequencing also established the nature of the homoeologous recombinants between the A, B and D-genomes of wheat identified with multi-colour GISH, i.e. which linkage groups were involved (Figs. 3c and d, Table 5S).

Recent papers have considered *Ae. mutica* to be closely related to *Ae. speltoides* and thus part of the B-genome lineage (Edet et al. 2018; Glémin et al. 2019; Bernhadt et al. 2020) and hence *Ae. mutica* was expected to recombine most frequently with the B-genome. Previous analysis of the 51 doubled haploid lines showed approximately equal recombination between *Ae. mutica* and the B- and D-genomes of wheat but no recombination with the A-genome (King et al. 2019). The D-genome is proposed to have arisen via a homoploid hybrid speciation between the A- and B-genome lineages (Marcussen et al. 2014; Li et al. 2022), possibly explaining the recombination originally observed between *Ae. mutica* and the B- and D-genomes of wheat. The analysis of the combined 119 lines, however, showed *Ae. mutica* to have recombined most frequently with the D-genome (75%) and least with the B-genome (7%). Recent studies on the evolution of wheat and its wild relatives have shown a far more complex evolution than previously thought, with *Ae. mutica* having a pivotal role (Bernhardt et al., 2020; Glémin et al. 2019). Both theories thus propose a close relationship between *Ae. mutica* and all three wheat genomes. The frequency of recombination between *Ae. mutica* and the B-genome of wheat was thus much lower than expected especially considering the close relationship between *Ae. mutica* and *Ae. speltoides*.

Recombination between the homoeologous chromosomes of wheat and a wild relative is usually suppressed by the presence of the *Ph1* gene. However, several wild relatives have been reported to carry a gene(s) able to suppress *Ph1* or alternatively promote homoeologous recombination even in the presence of *Ph1*. These include *Aegilops umbellulata* (Riley et al., 1960), *Thinopyrum elongatum* (Dvorak et al., 1987), *Ae. mutica* (Riley and Dover, 1972a and b), *Ae. speltoides* (Dvorak et al. 2006), and *Aegilops geniculata* (Koo et al. 2017). The homozygous introgression lines generated by the WRC at Nottingham were generated in the presence of *Ph1*, thus confirming the presence of a *Ph1* suppressor/homoeologous recombination promotor in *Ae. mutica* previously reported by Dover and Riley, (1972a and b). Homoeologous recombination both between heterozygous introgressions and the wheat genome (Table 2) and within the wheat genome (Table 5S) also occurred during gamete formation in later backcross generations (Fig. 5a). This later recombination was found in six of the seven T chromosomes—2, 3, 4, 5, 6 and 7 (Table 2). Dover and Riley (1972a and b) reported that *Ae. mutica* possessed two different *Ph1* suppressor/homoeologous recombination promotor loci. A similar situation has been observed in *Ae. speltoides* where two *Ph1* suppressor loci were identified, located on chromosomes 3S and 7S with a third minor locus on linkage group 5S (Dvorak et al 2006). However, with the levels of further recombination seen in all but one of the *Ae. mutica* chromosomes, it is not possible at this stage to identify the location of the promotors. Further work is being undertaken to ascertain the nature of homoeologous recombination observed between chromosomes of wheat and *Ae. mutica*.

Skim-sequencing confirmed the presence of introgressions identified through KASP genotyping and multi-colour GISH. However, it was also able to show that some lines carrying introgressions from different BC_1_ plants, i.e. generated via independent recombination events, had almost identical sites of recombination in both wheat and *Ae. mutica* (Fig. 4; Table 6S). Recombination hotspots have been observed in many species, including wheat (Faris et al. 1999; Avni et al. 2014; Choulet et al. 2014), with the number of hotspots related to chromosome length (He et al. 2017). The hotspots are thought to occur in gene-rich regions of the genome, being particularly associated with genes mostly expressed during meiosis (Darrier et al. 2017). The newly developed genome assembly of *Ae. mutica* (Grewal et al. 2025) may help to elucidate the genes present in the hotspots in *Ae. mutica* as compared to those present in the equivalent hotspots in wheat.

The majority of the introgressions observed between *Ae. mutica* and wheat were due to recombination in the distal or sub-telomeric regions of the chromosomes. This was as expected as preferential recombination is known to occur in these regions of wheat chromosomes (Dvorak and Chen 1984; Lukaszewski and Curtis 1993; Gill et al. 1996; Akhunov et al. 2003). However, recombination was also found to have occurred in pericentromeric regions of the wheat chromosomes (Fig. 5b and c). Pericentromeric recombination has been observed in previous studies on wheat/wild relative introgressions, both in newly generated introgressions e.g., between wheat and *Thinopyrum elongatum* (Baker et al. 2020; Zhang et al. 2020) and in older established introgressions (Heuberger et al. 2024).

Preliminary studies on the 68 new introgression lines developed from *Ae. mutica* have already demonstrated the potential of this underutilised species to carry new and potentially exciting genetic variation for a wide range of traits including both abiotic and biotic stresses (Fellers et al. 2020) and beneficial grain mineral content (Guwela et al. 2024). In addition, several of these lines have been screened over the past five years by UK commercial wheat breeders as part of the Breeders Observation Panel, which was funded by the BBSRC Developing Future Wheat Programme. Many useful traits have been identified in these lines through this collaborative effort (unpublished data) that are being utilised in pre-breeding programmes in these breeding companies. Seed of 48 of the *Ae. mutica* introgression lines have been deposited at the GRU at the JIC, along with all the *Ae. mutica* DH lines. The remaining 25 lines will be deposited as soon as possible after multiplication. All of these lines are therefore free of IP either from the GRU or from Nottingham.

## Supplementary Information

Below is the link to the electronic supplementary material.

Supplementary file1 (PDF 381 KB)

Supplementary file2 (DOCX 29524 KB)

Supplementary file3 (PDF 51 KB)

Supplementary file4 (XLSX 123 KB)

## Data Availability

The raw skim-sequence reads for all wheat-*Ae. mutica* introgression lines have been deposited at the European Nucleotide Archive (ENA) under project accession PRJEB89936. The raw genotyping data is available from the authors on request.

## Code availability

The custom scripts for the combined introgression mapping pipeline are available via https://github.com/Surbhigrewal/Introgression_mapping.
