# Supplementary material for: The transfer of 98% of the genome of Aegilops mutica into wheat (Triticum aestivum)
Source: Theor Appl Genet. 2026 Feb 9;139(2):65. doi: 10.1007/s00122-026-05173-1 (PMC12886214; doi:10.1007/s00122-026-05173-1)
Supplement: Supplementary file 1 — Supplementary file1 (PDF 381 KB) [file 122_2026_5173_MOESM1_ESM.pdf]

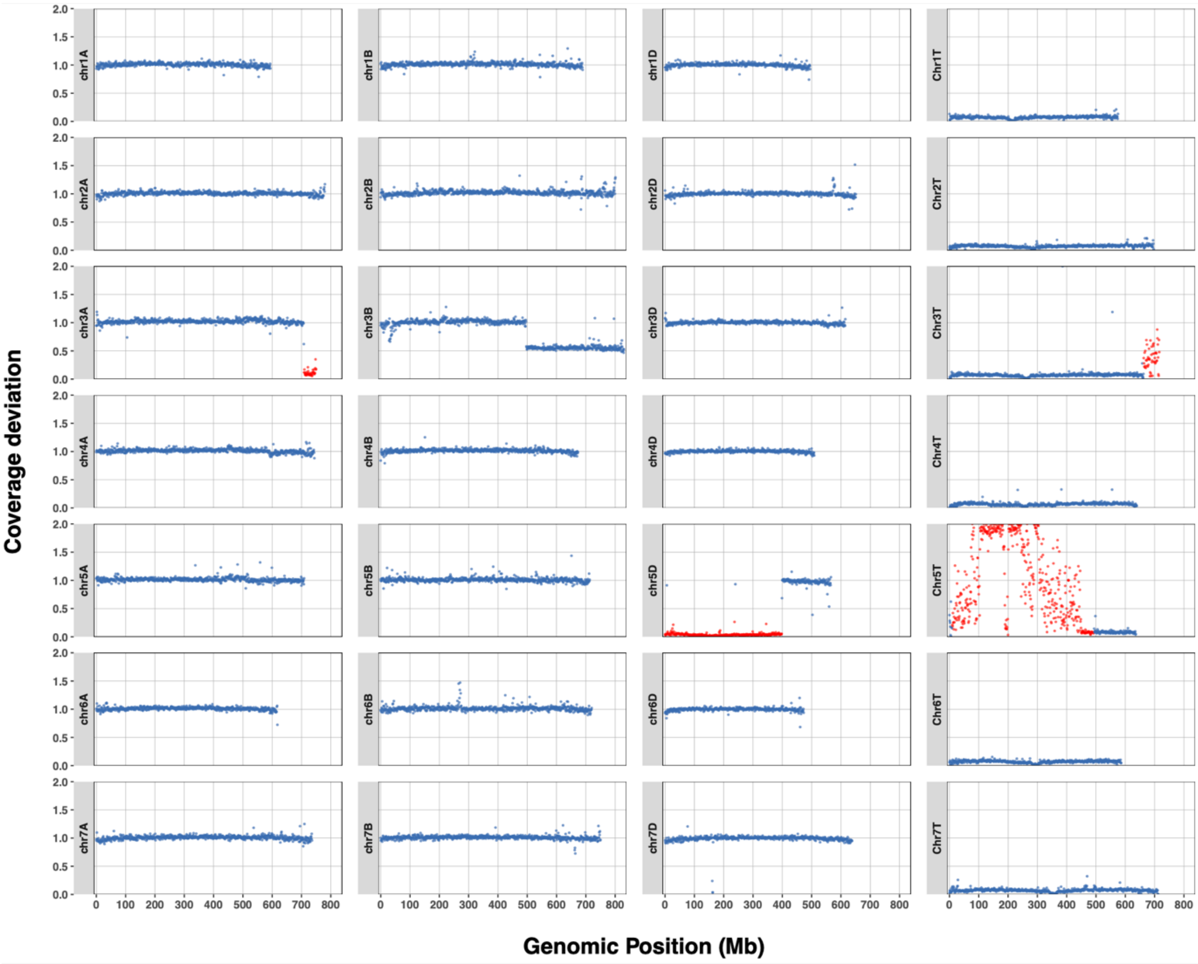

Fig. 1S Coverage deviation analysis using a skim-sequencing plot of introgression line Mut27. The plot showing two complete drops in read coverage on the wheat chromosomes 3A and 5D (red dots) due to the corresponding presence of *Ae. mutica* introgressions on chromosomes 3T and 5T indicated by increase in read coverage (red dots). The absence of an increase in read coverage of *Ae. mutica* chromosome 3T, apart from the small region at its end, indicates the drop in wheat coverage on chromosome 3B is likely due to a wheat deletion in one copy of 3B rather than the presence of an *Ae. mutica* introgression.
