# Supplementary material for: The transfer of 98% of the genome of Aegilops mutica into wheat (Triticum aestivum)
Source: Theor Appl Genet. 2026 Feb 9;139(2):65. doi: 10.1007/s00122-026-05173-1 (PMC12886214; doi:10.1007/s00122-026-05173-1)

**Supplementary Figure 2.** Cytogenetic characterisation of the 68 new *Ae. mutica* introgression lines reported in this paper. Lines shown:

WRC21_Mut2 WRC21_Mut39

WRC21_Mut3 WRC21_Mut40

WRC21_Mut4 WRC21_Mut41

WRC21_Mut5 WRC21_Mut42

WRC21_Mut6 WRC21_Mut43

WRC21_Mut7 WRC21_Mut44

WRC21_Mut10 WRC21_Mut45

WRC21_Mut12 WRC21_Mut46

WRC21_Mut13 WRC21_Mut47

WRC21_Mut14 WRC21_Mut48

WRC21_Mut15 WRC21_Mut49

WRC21_Mut16 WRC21_Mut50

WRC21_Mut17 WRC21_Mut51

WRC21_Mut18 WRC21_Mut52

WRC21_Mut19 WRC21_Mut53

WRC21_Mut20 WRC21_Mut54

WRC21_Mut21 WRC21_Mut55

WRC21_Mut22 WRC21_Mut56

WRC21_Mut23 WRC21_Mut57

WRC21_Mut24 WRC23_Mut58

WRC21_Mut25 WRC23_Mut62

WRC21_Mut26 WRC23_Mut66

WRC21_Mut27 WRC23_Mut70

WRC21_Mut28 WRC23_Mut73

WRC21_Mut29 WRC23_Mut74

WRC21_Mut30 WRC23_Mut75

WRC21_Mut31 WRC23_Mut77

WRC21_Mut32 WRC23_Mut78

WRC21_Mut33 WRC23_Mut79

WRC21_Mut34 WRC23_Mut80

WRC21_Mut35 WRC23_Mut82

WRC21_Mut36 WRC23_Mut84

WRC21_Mut37 WRC23_Mut85

WRC21_Mut38 WRC23_Mut86


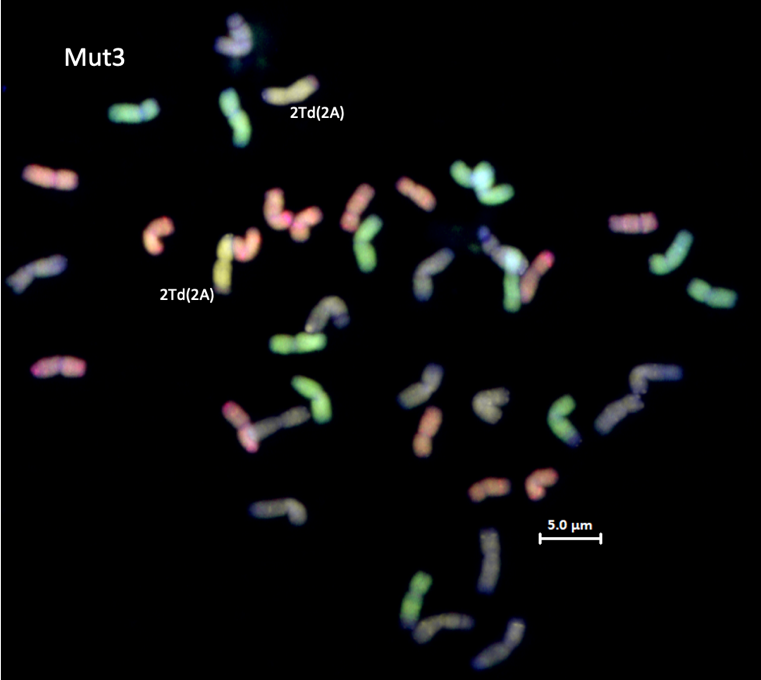

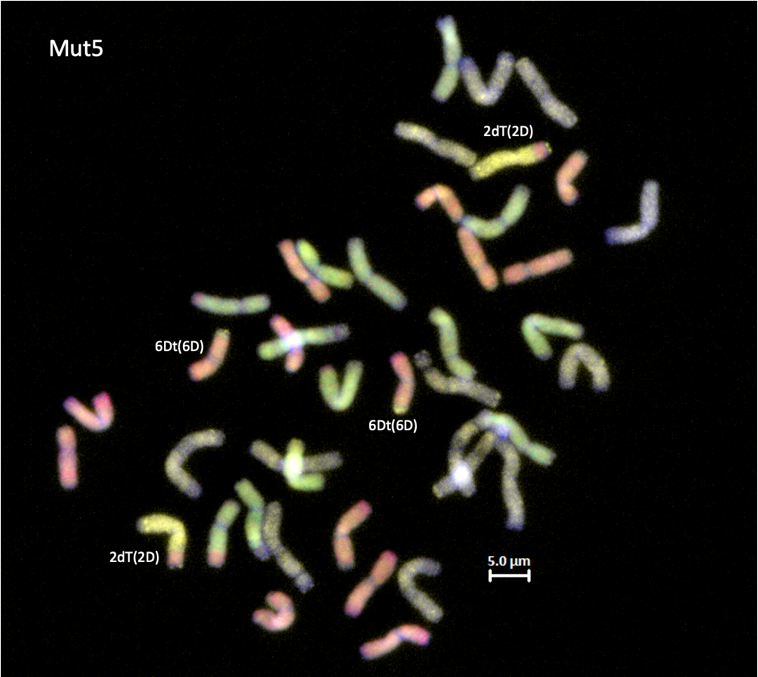

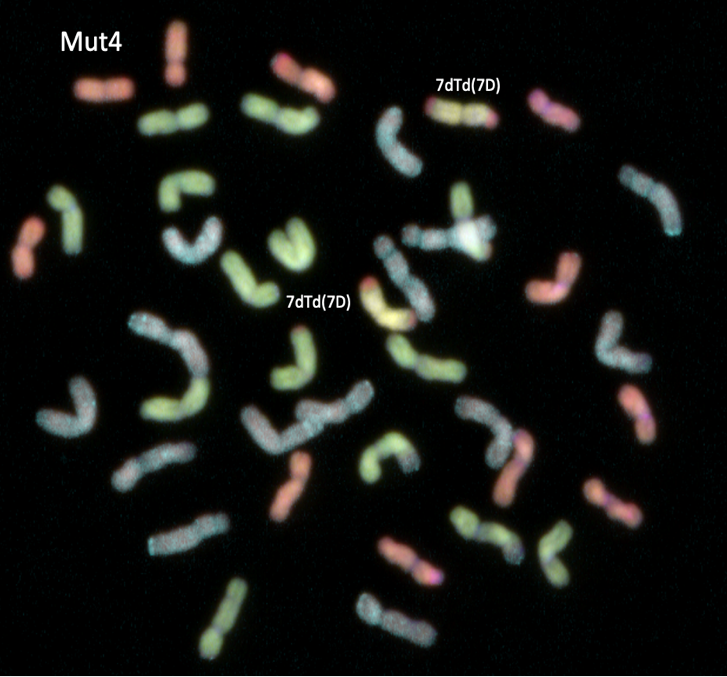

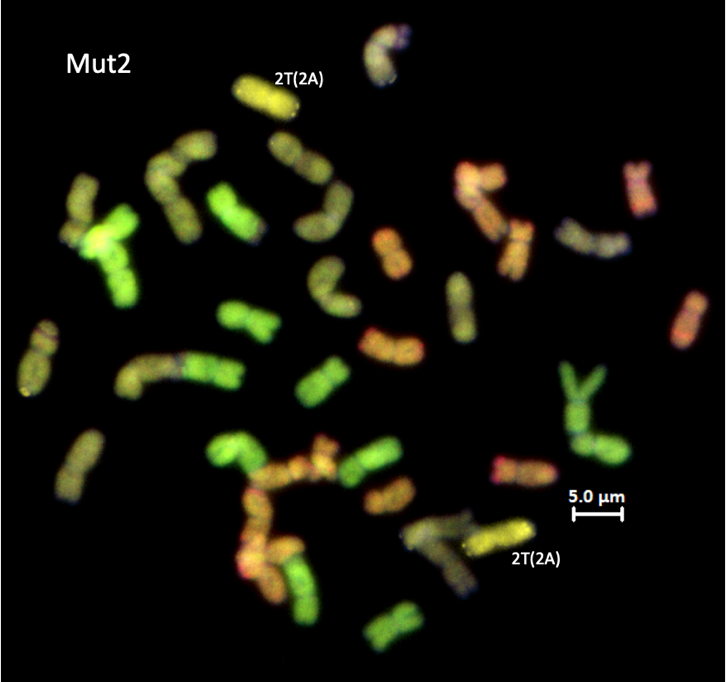

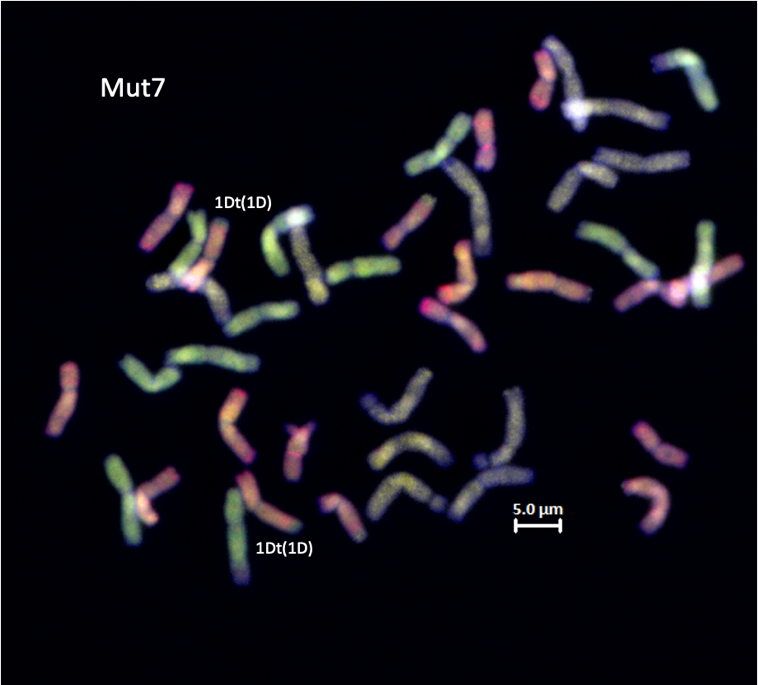

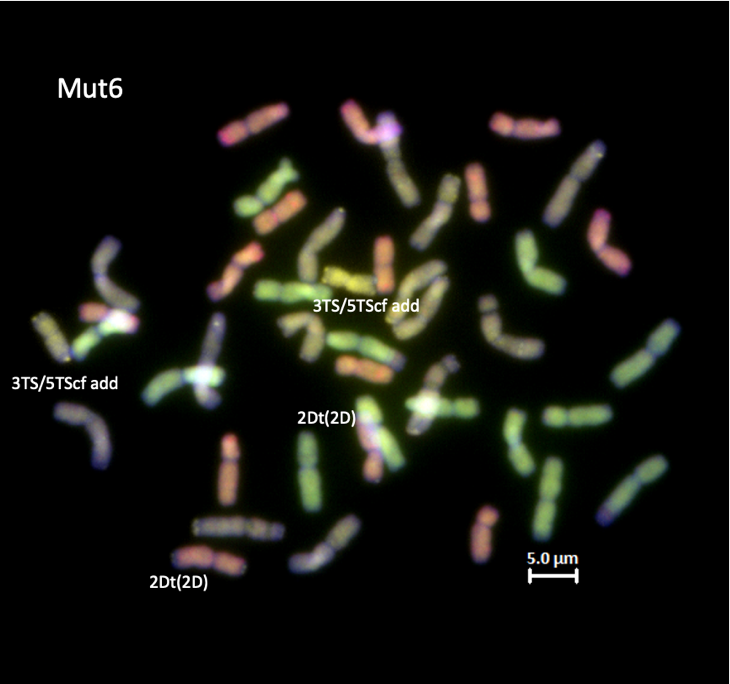


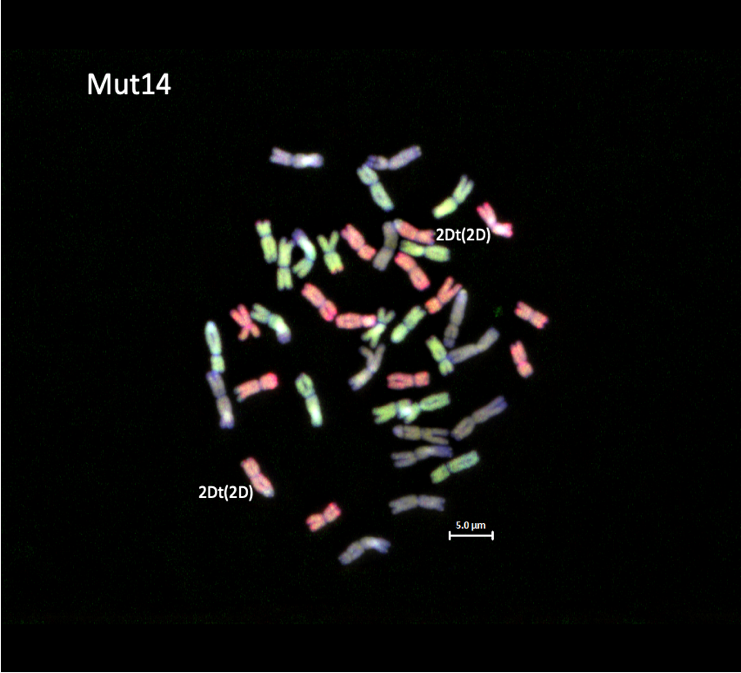

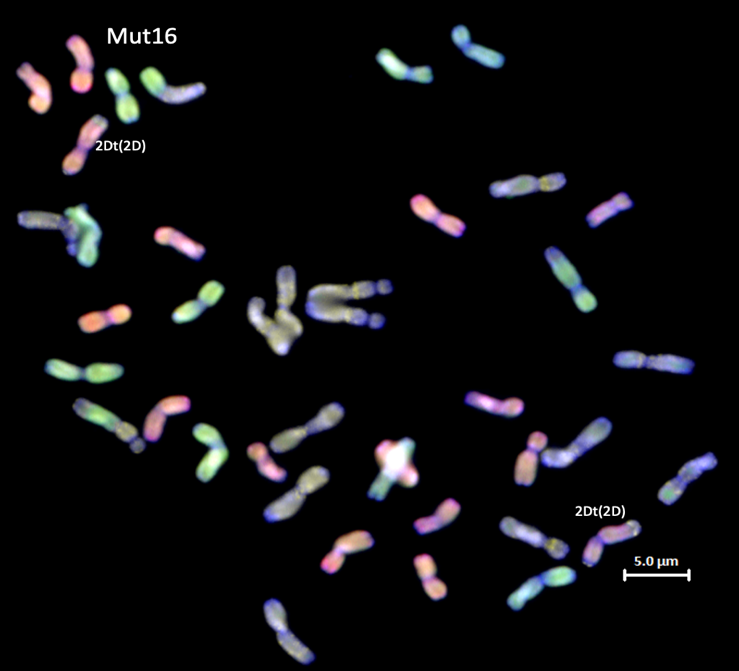

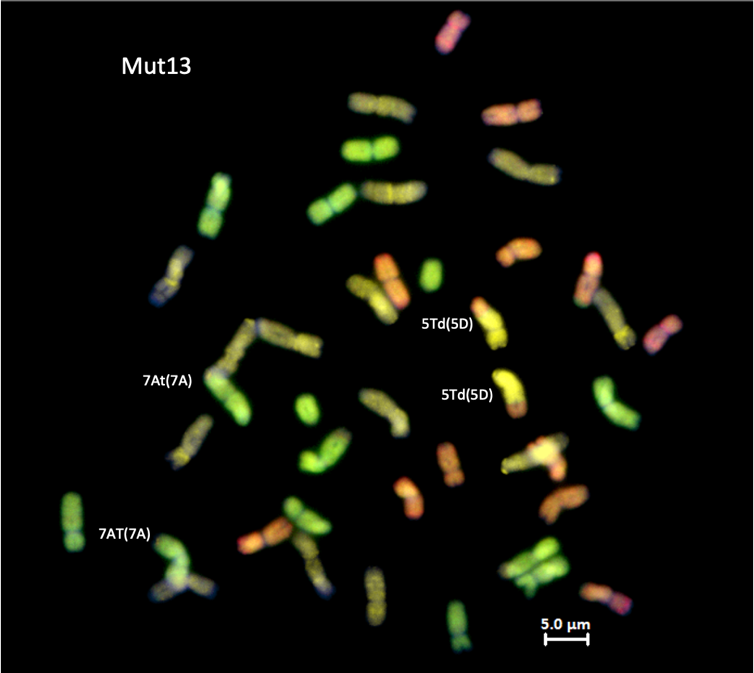

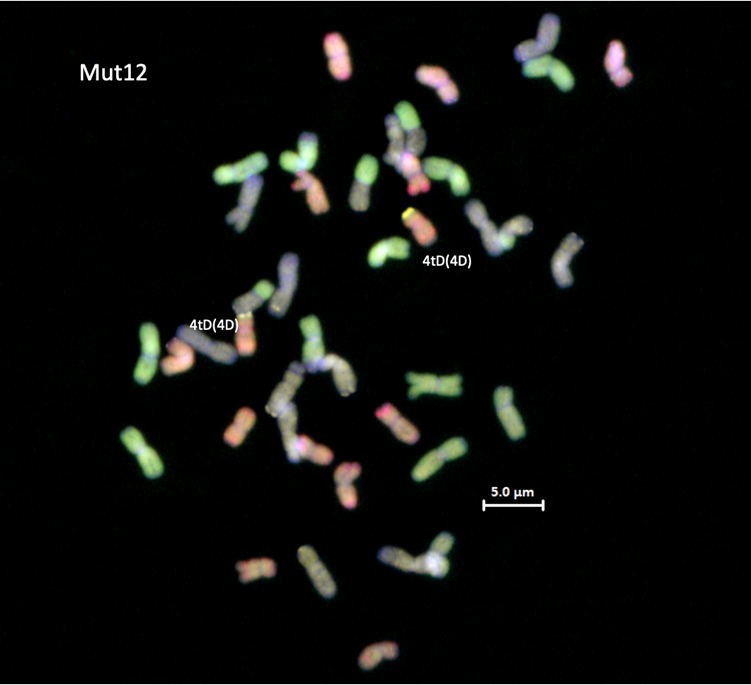

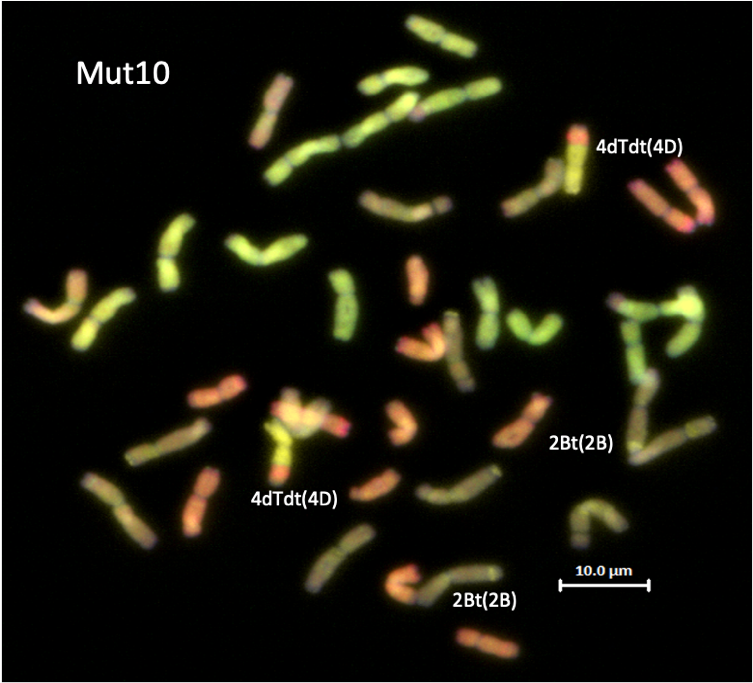

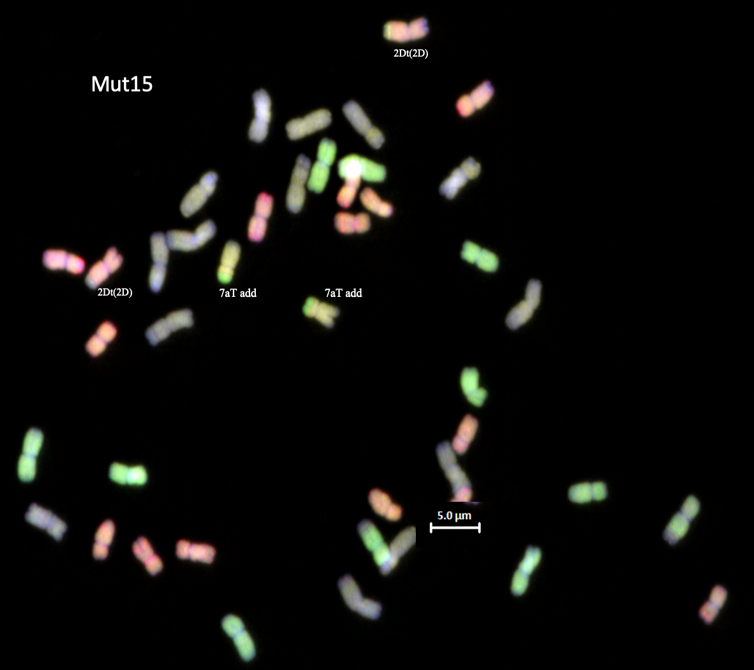


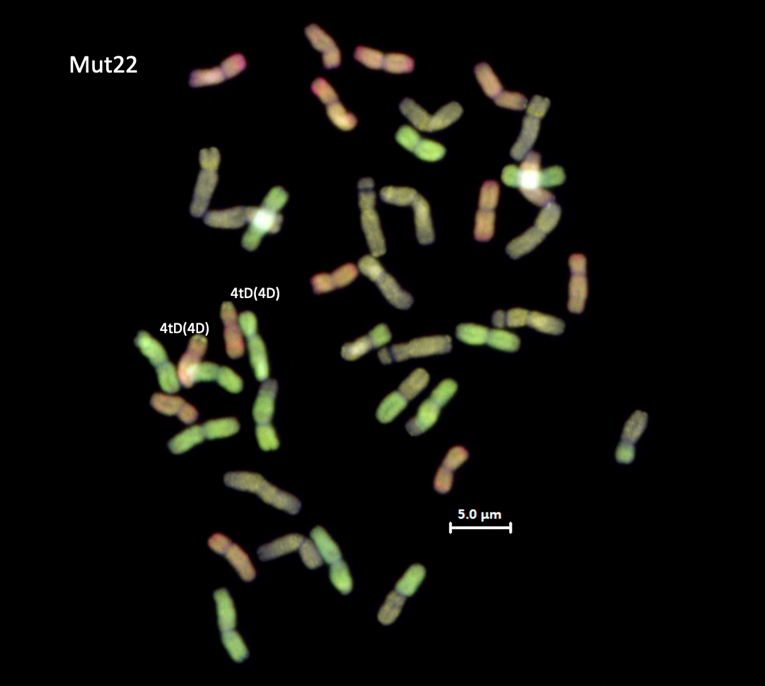

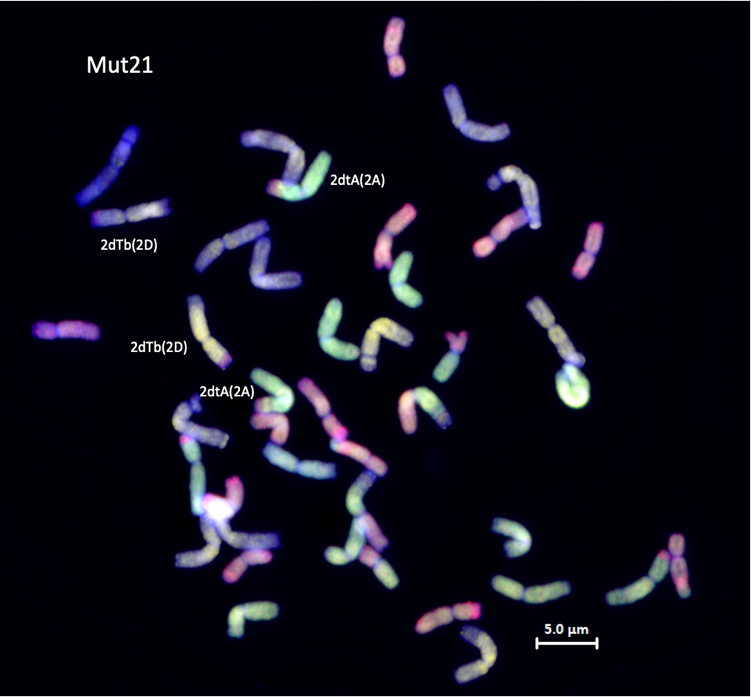

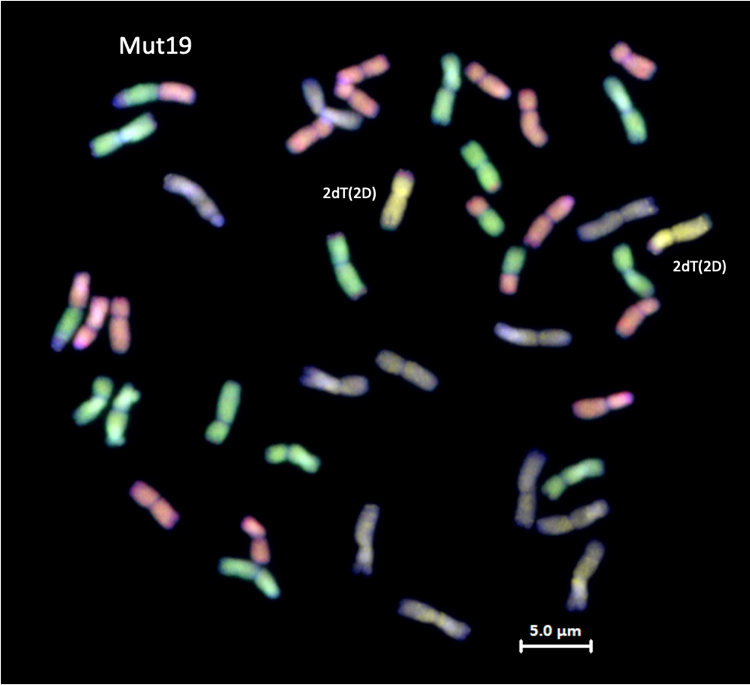

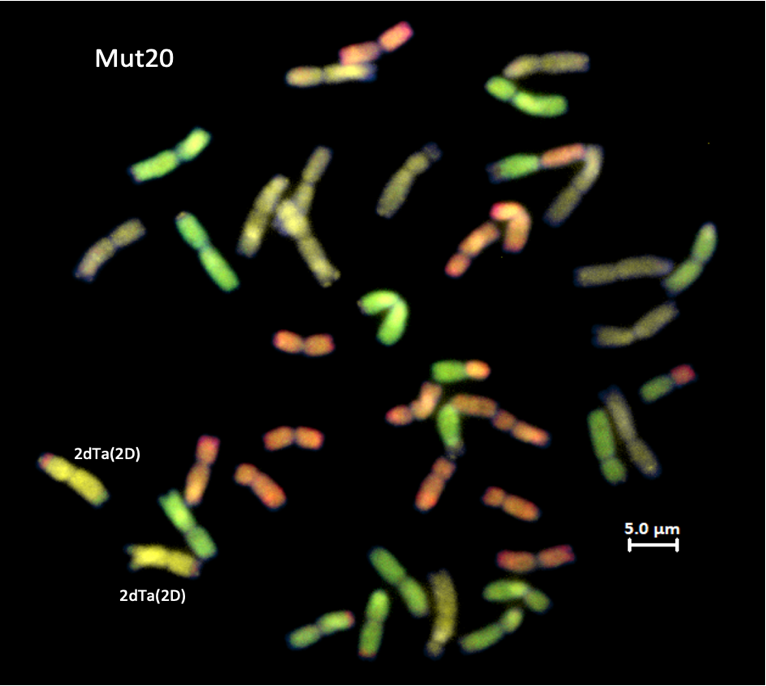

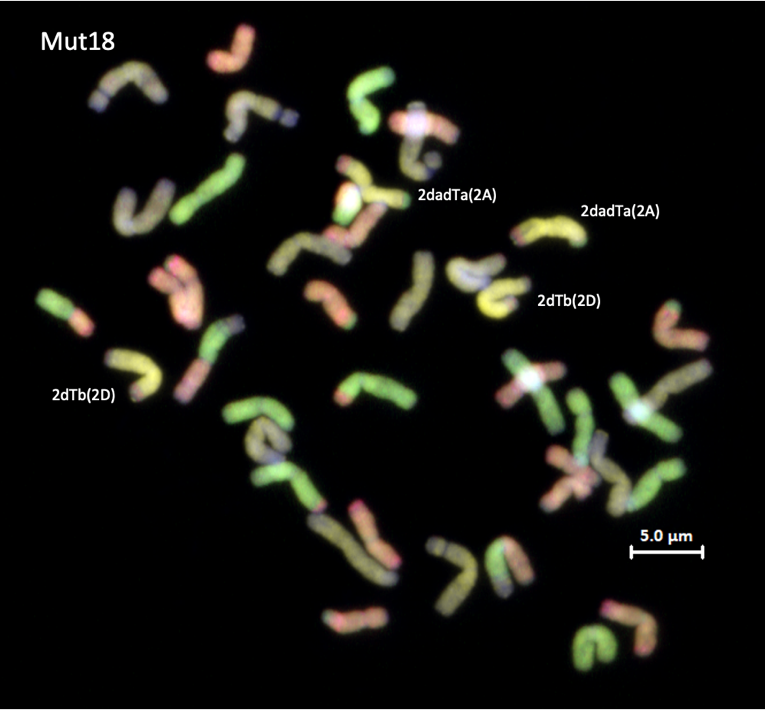

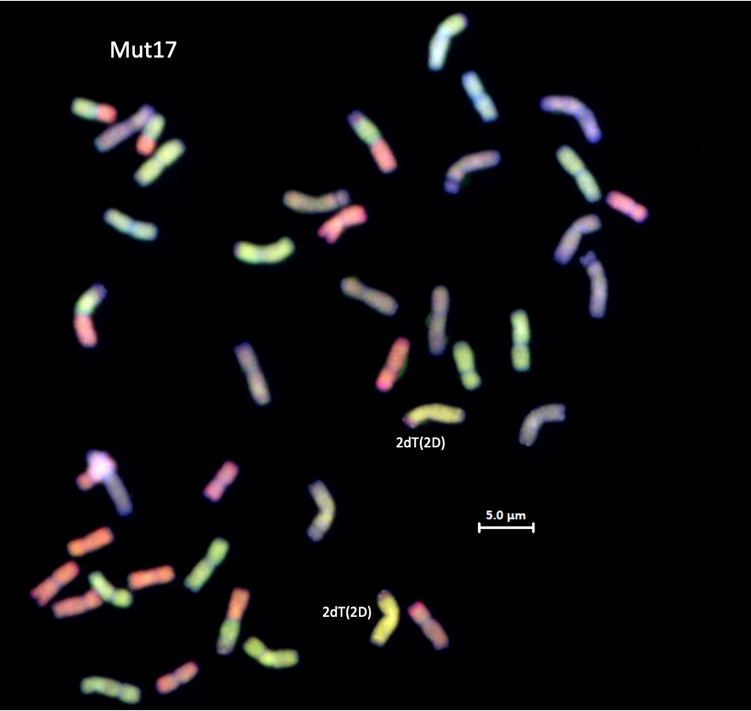


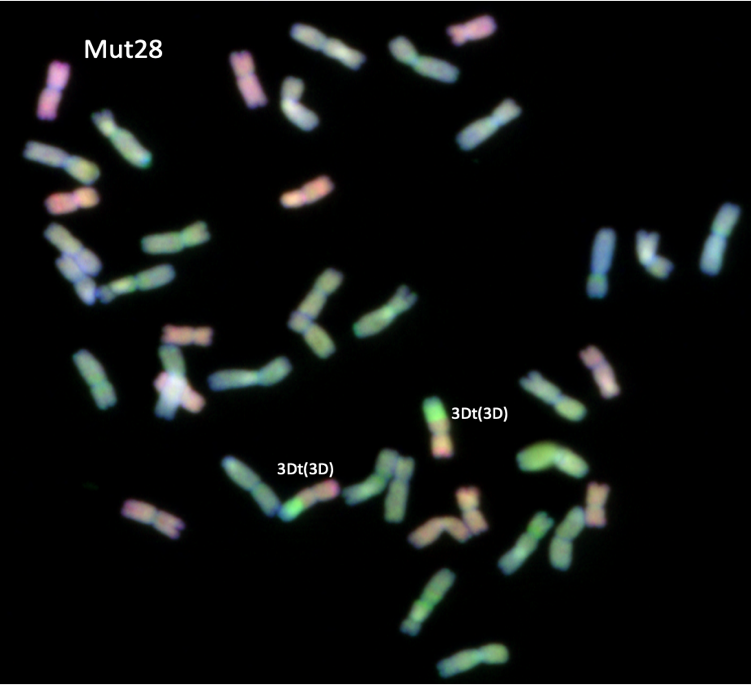

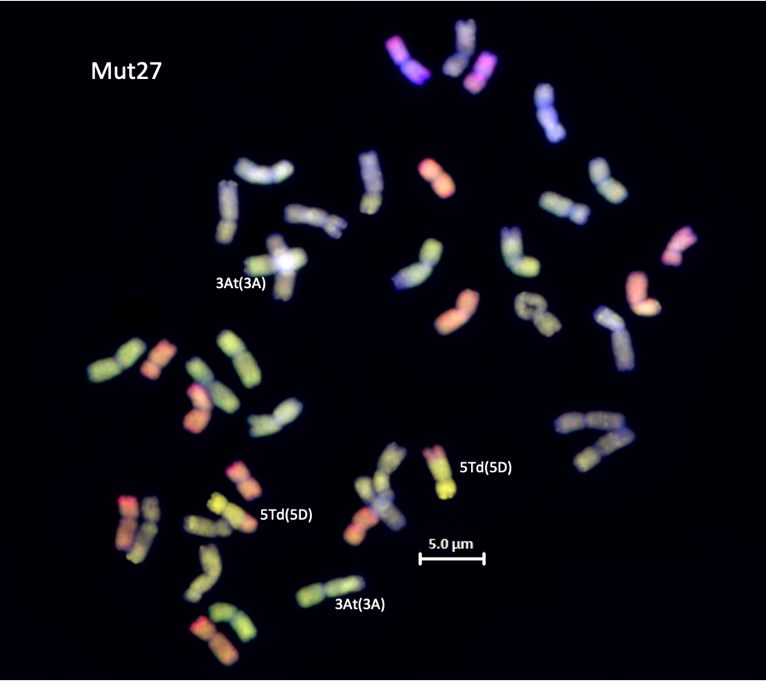

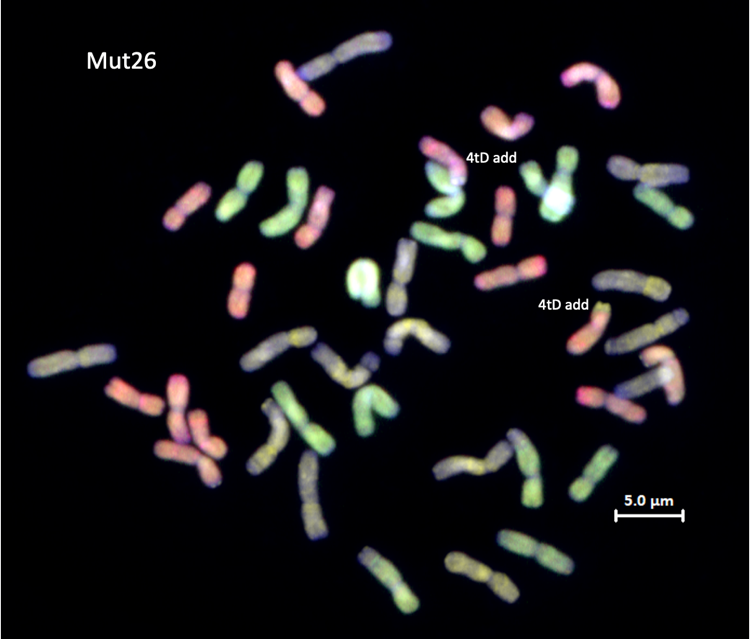

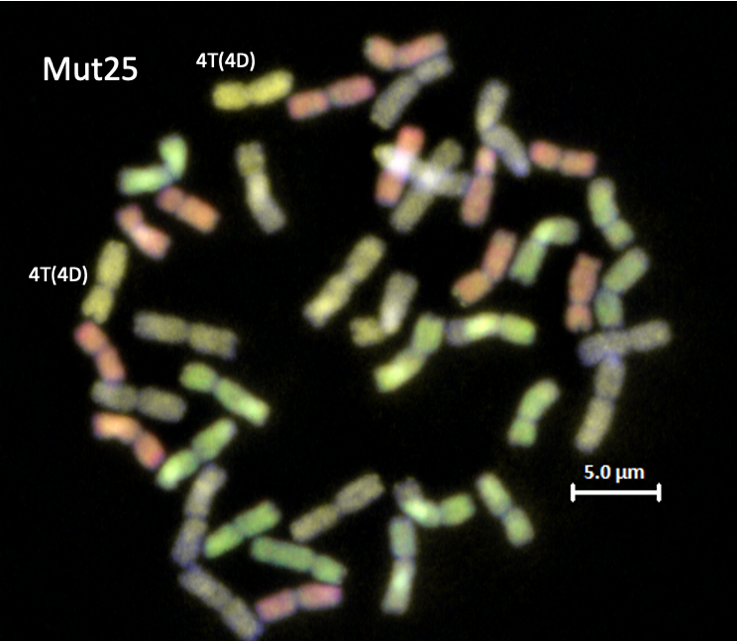

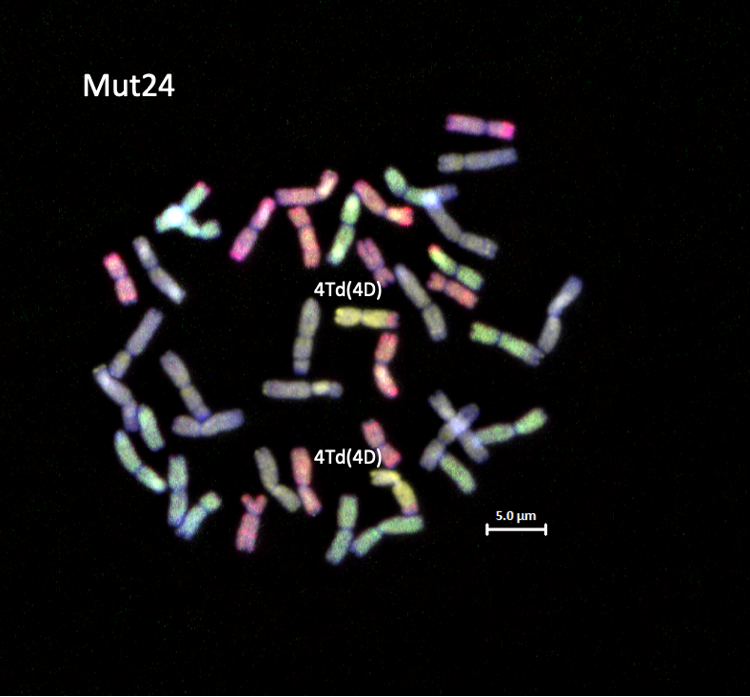

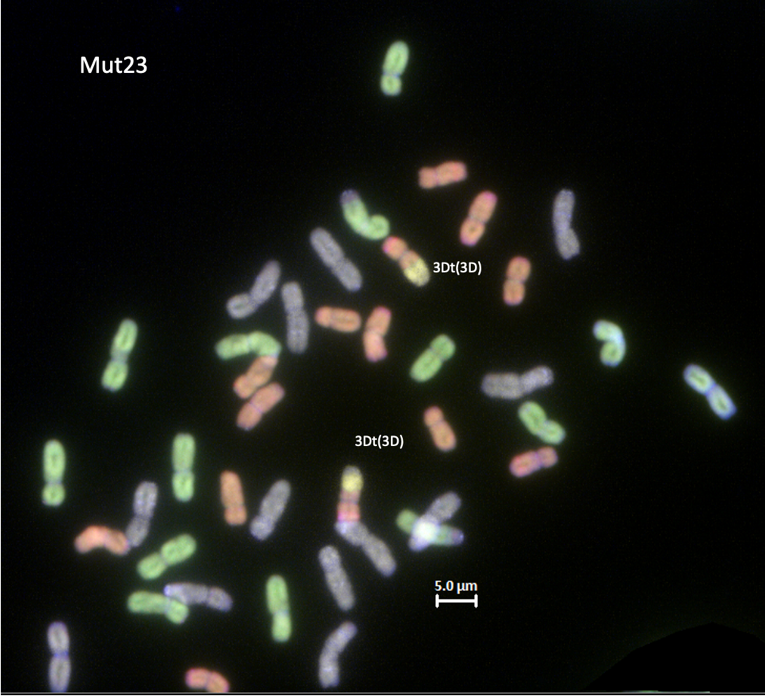


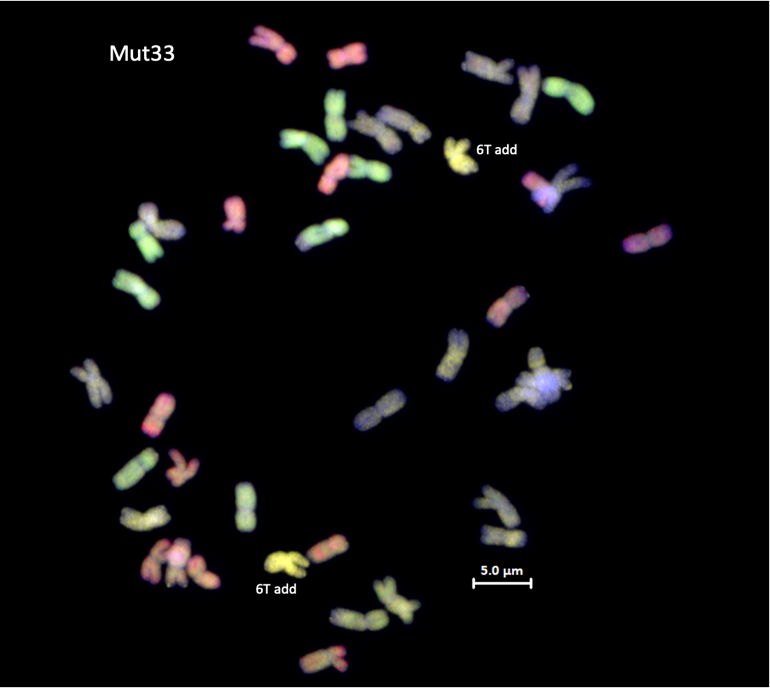

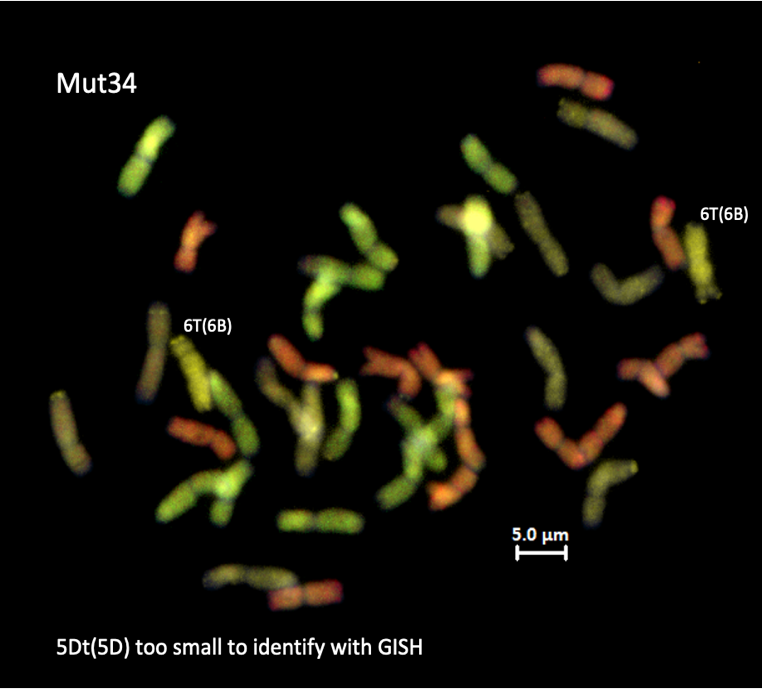

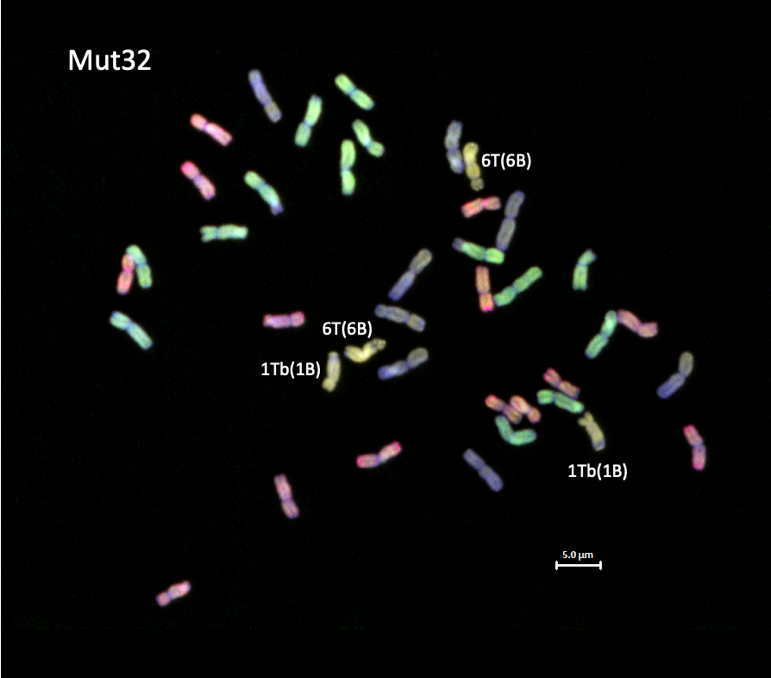

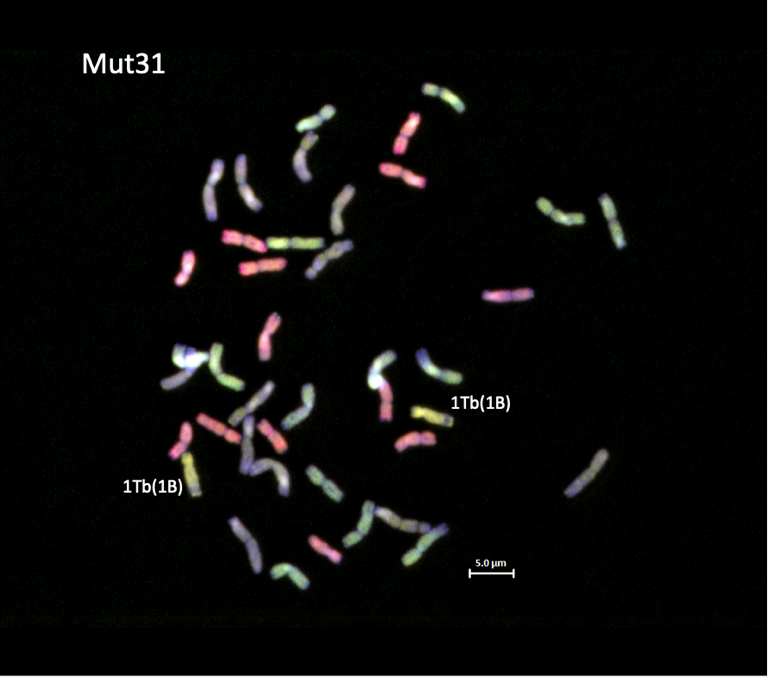

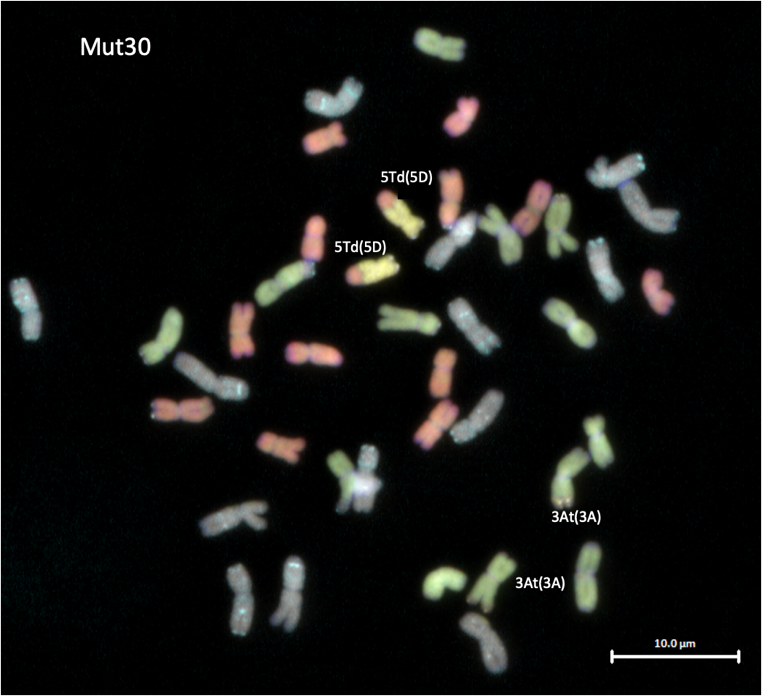

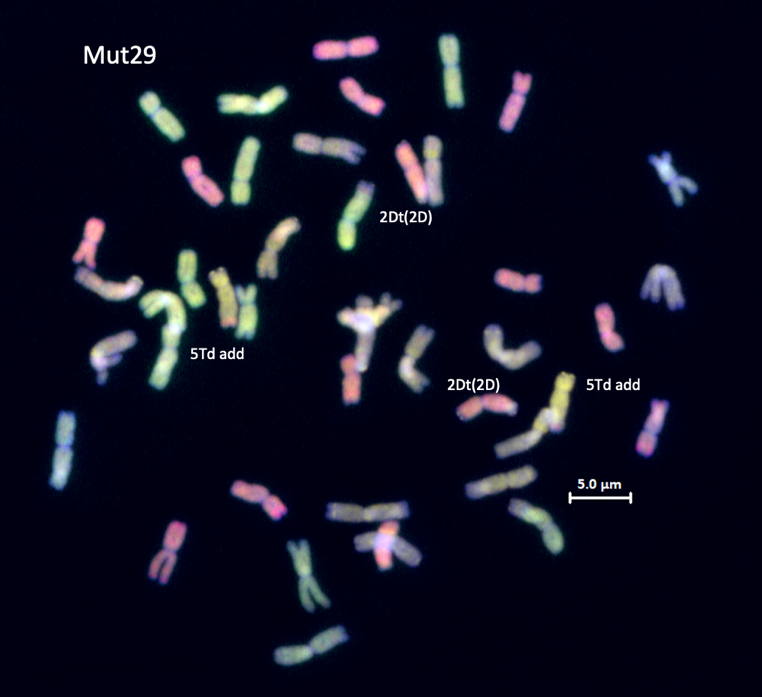


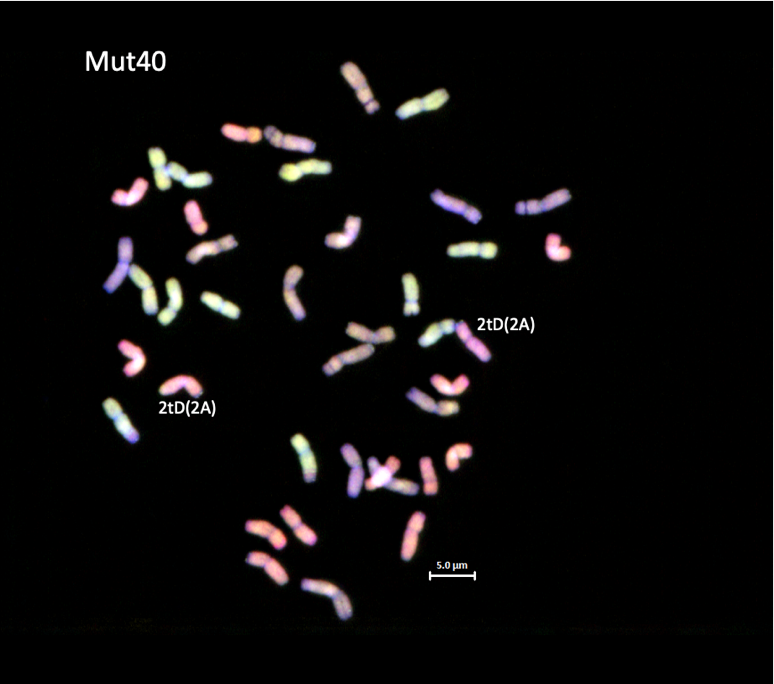

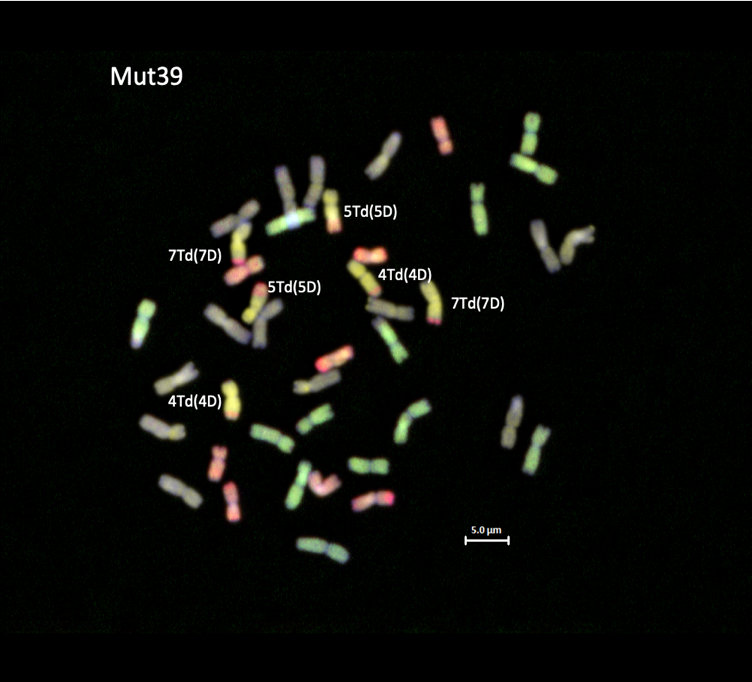

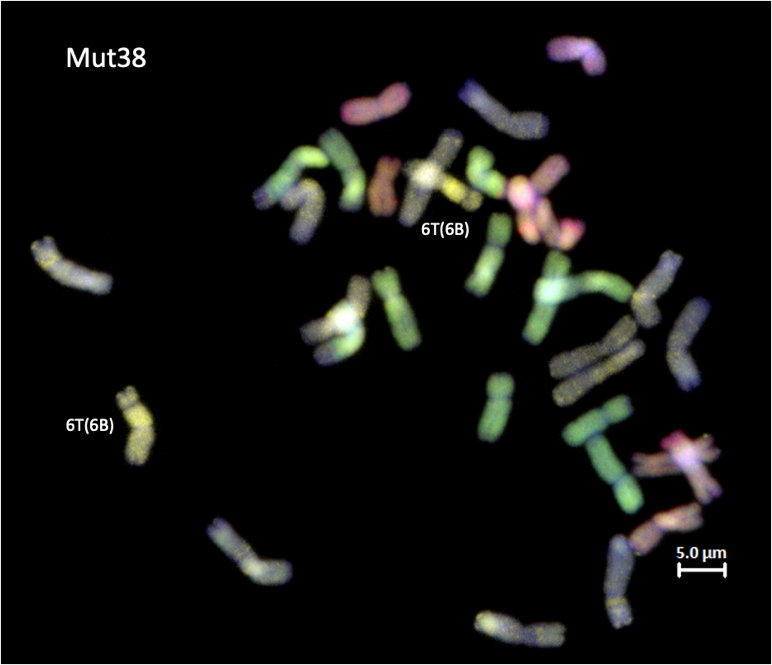

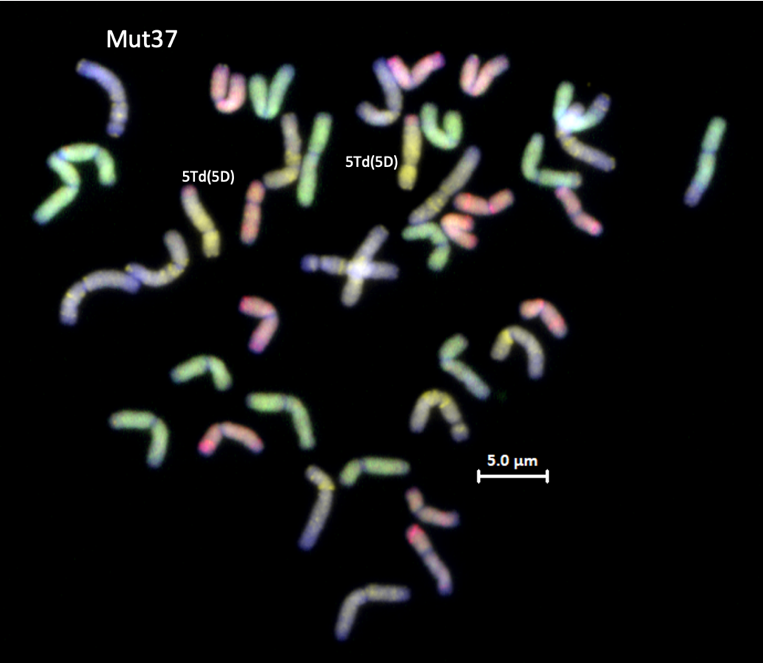

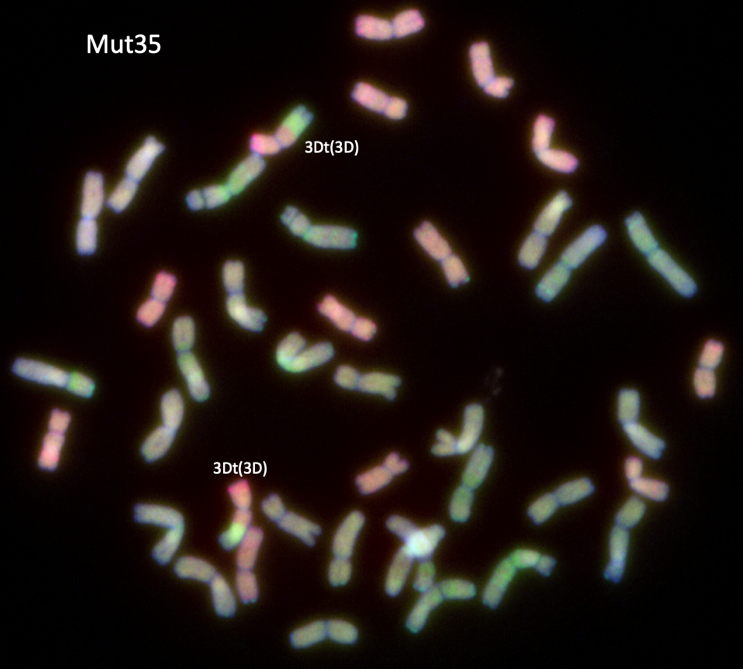

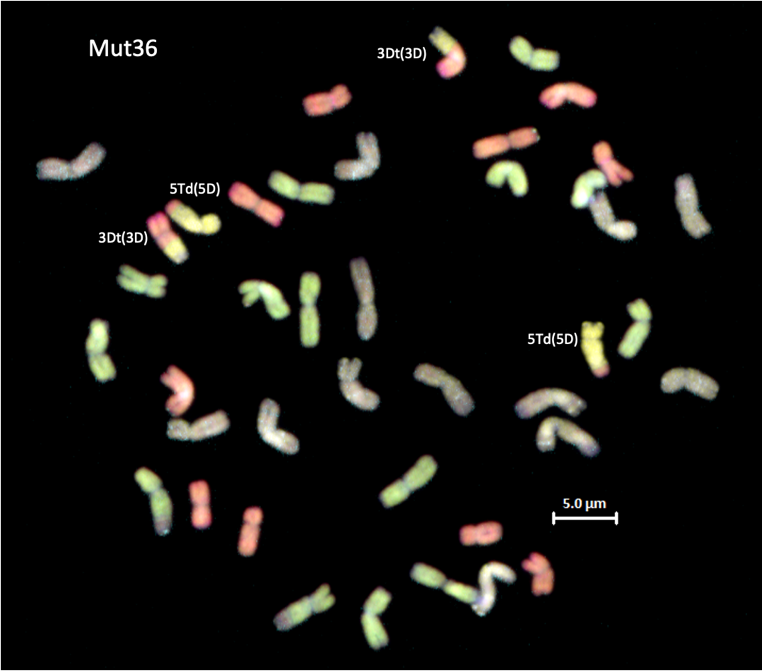


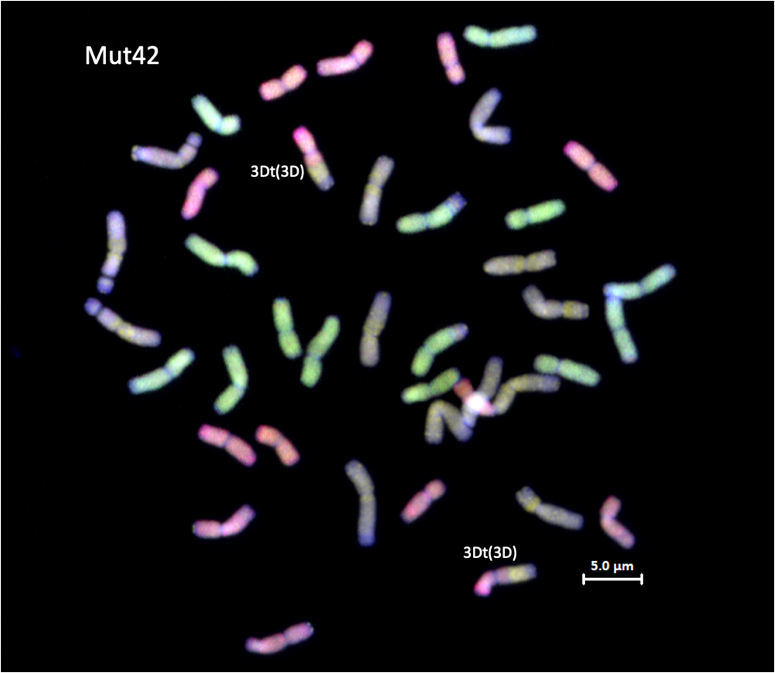

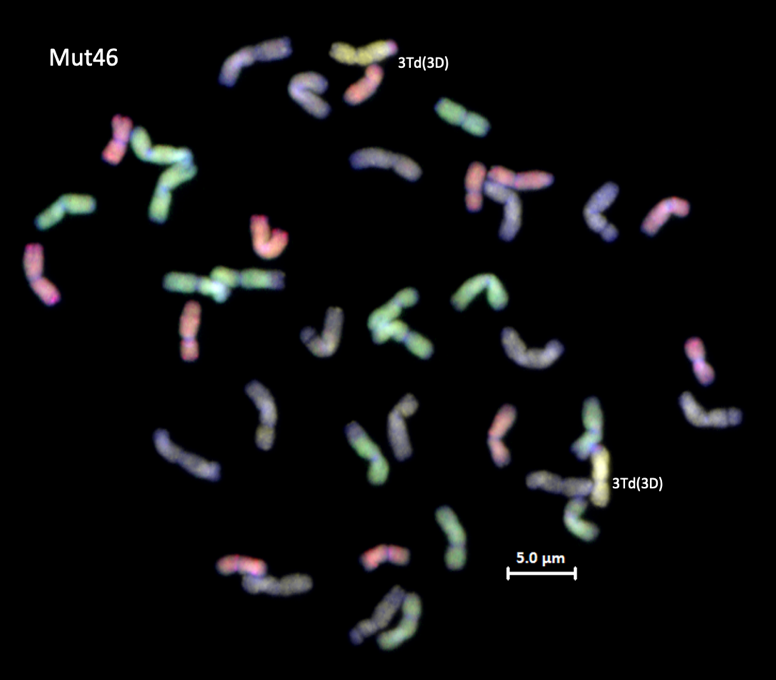

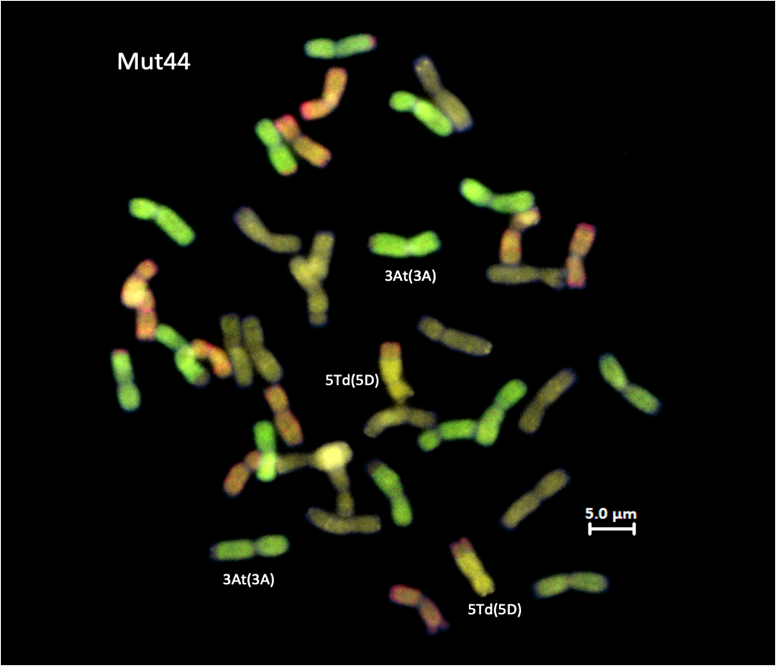

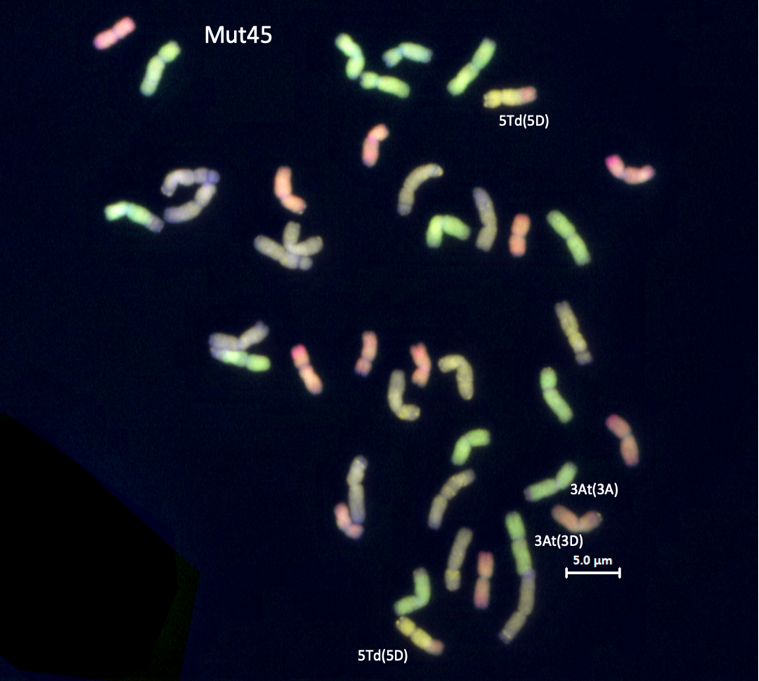

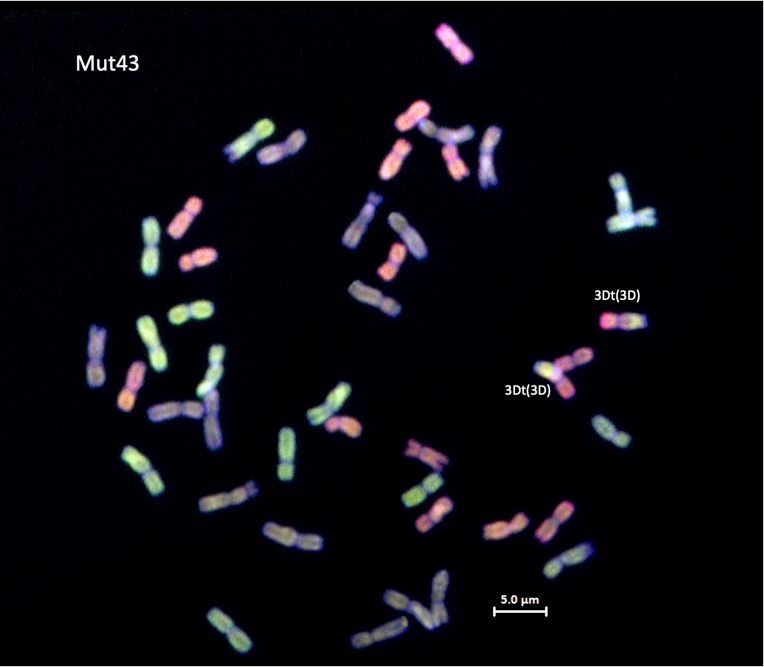

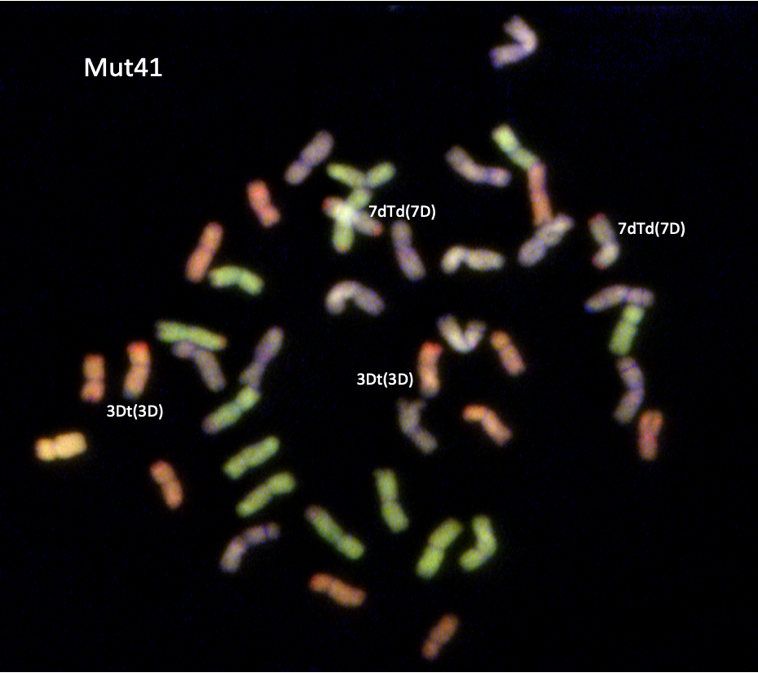


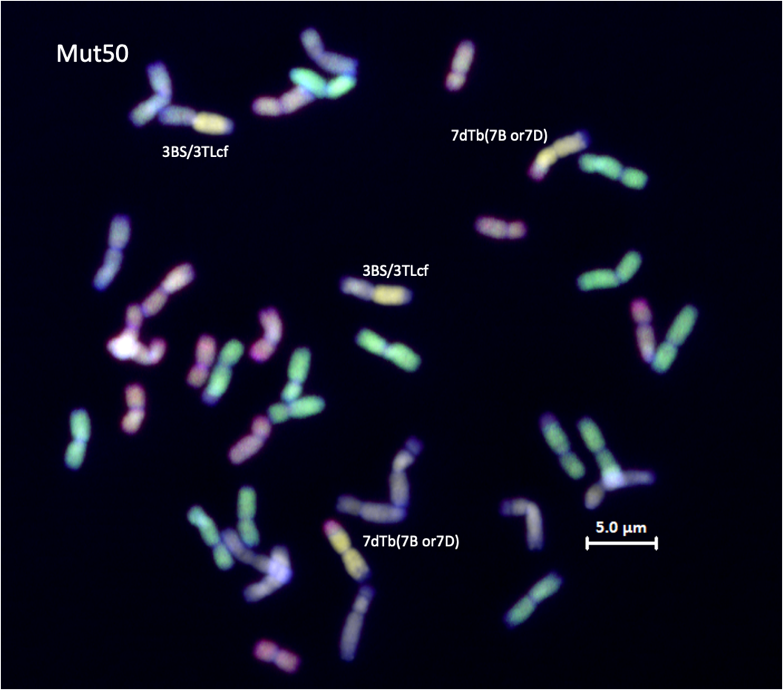

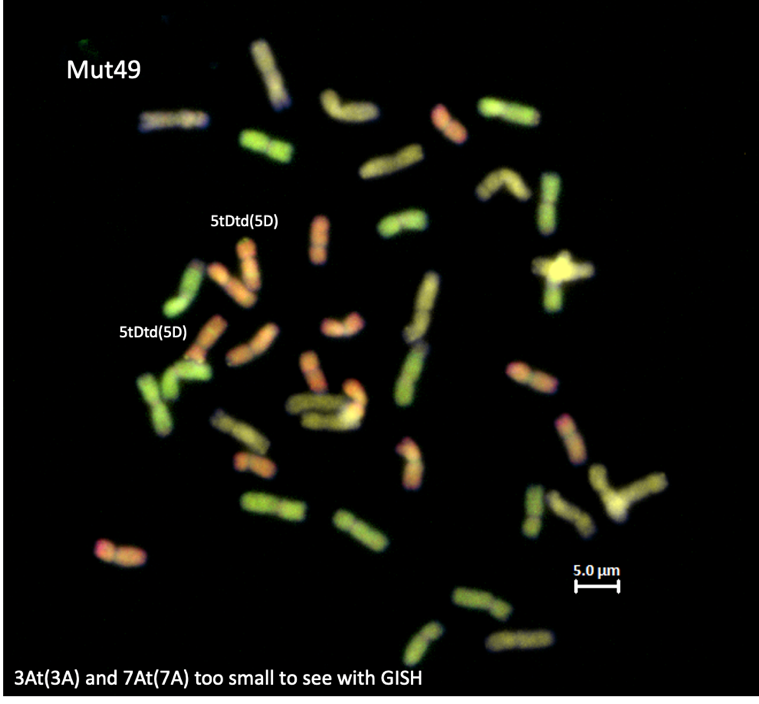

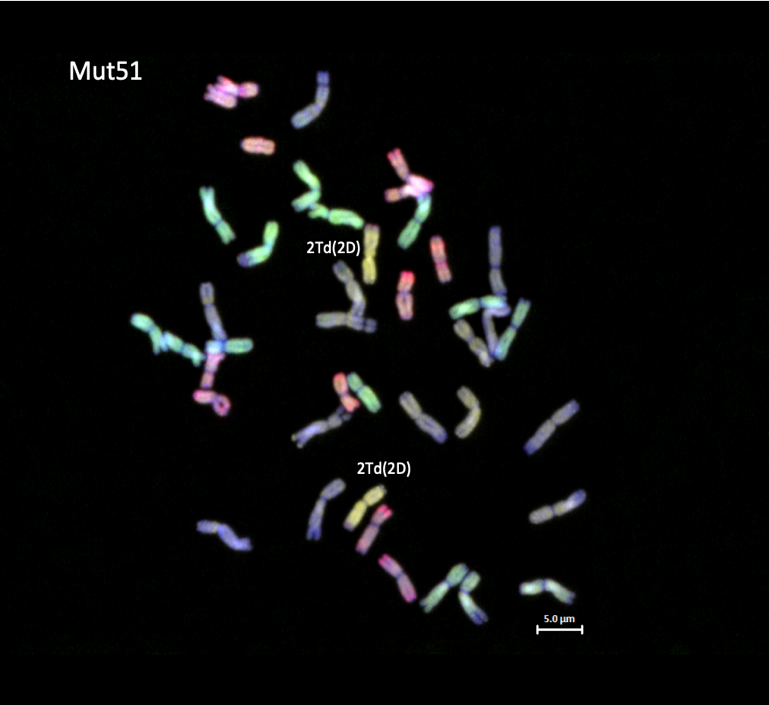

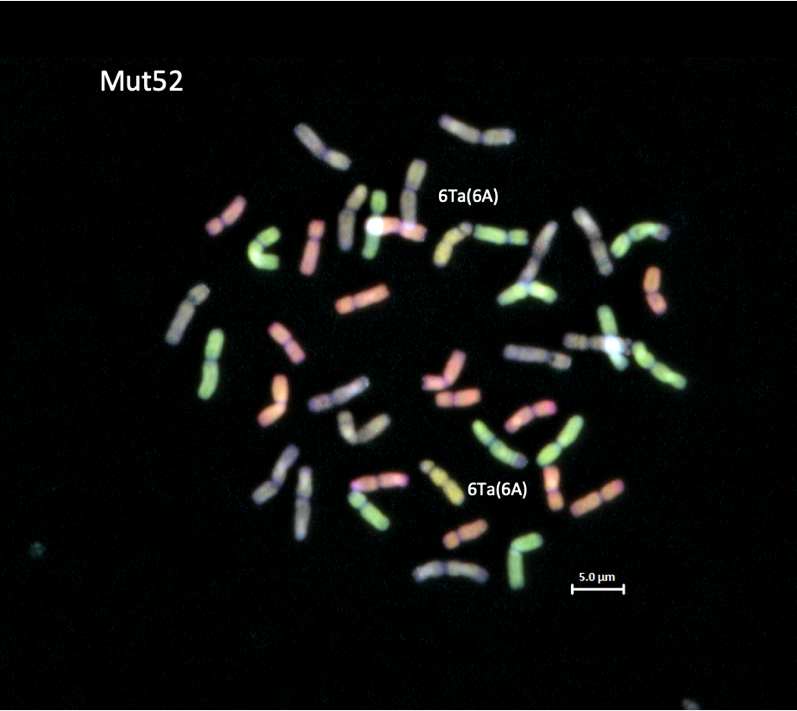

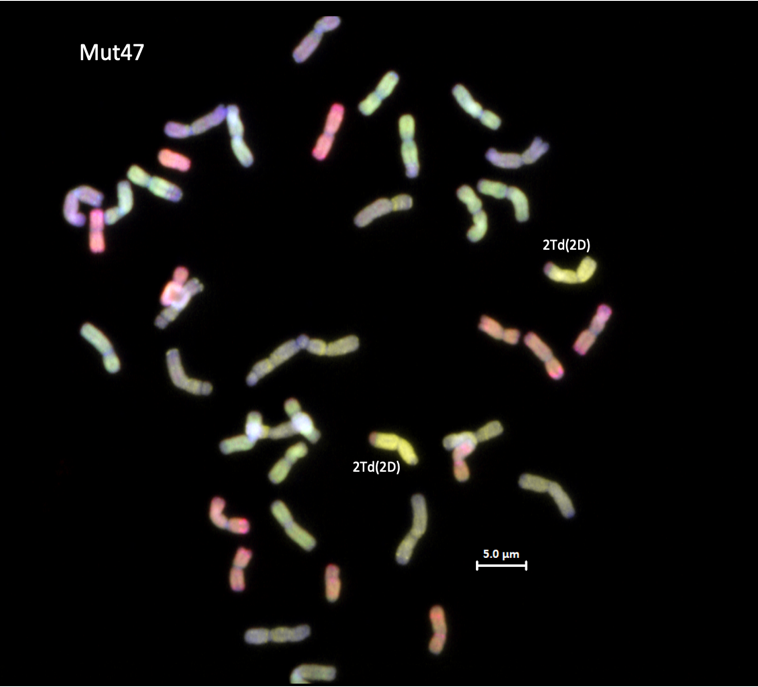

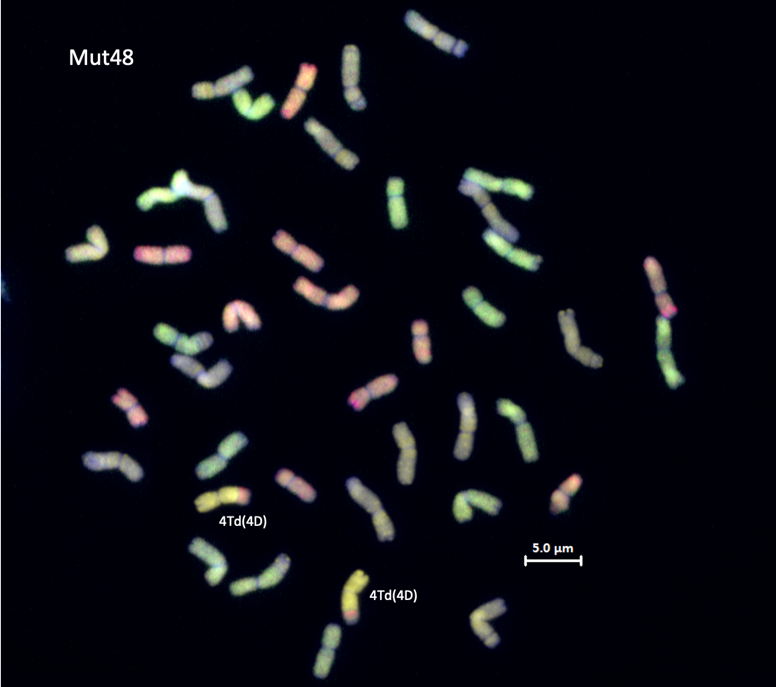


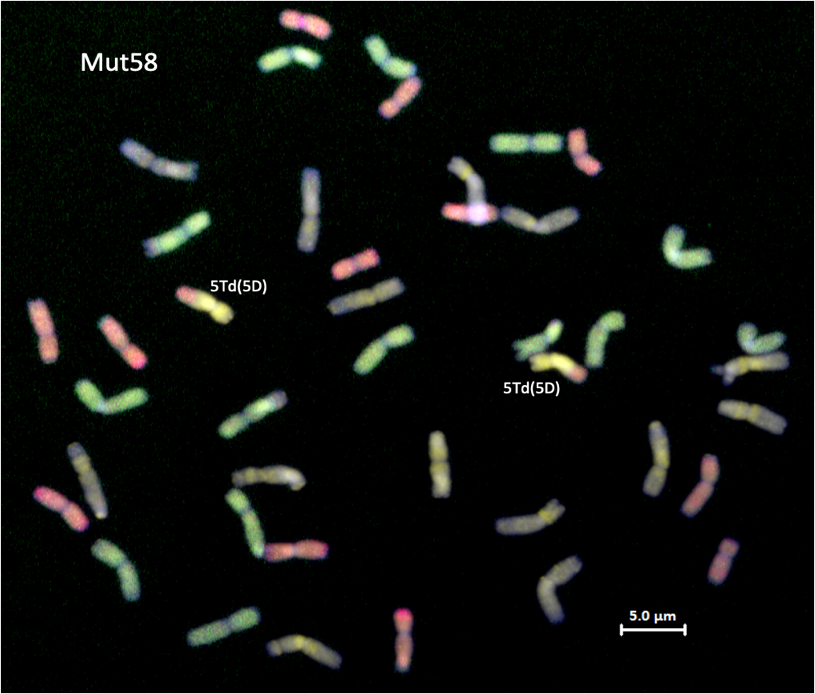

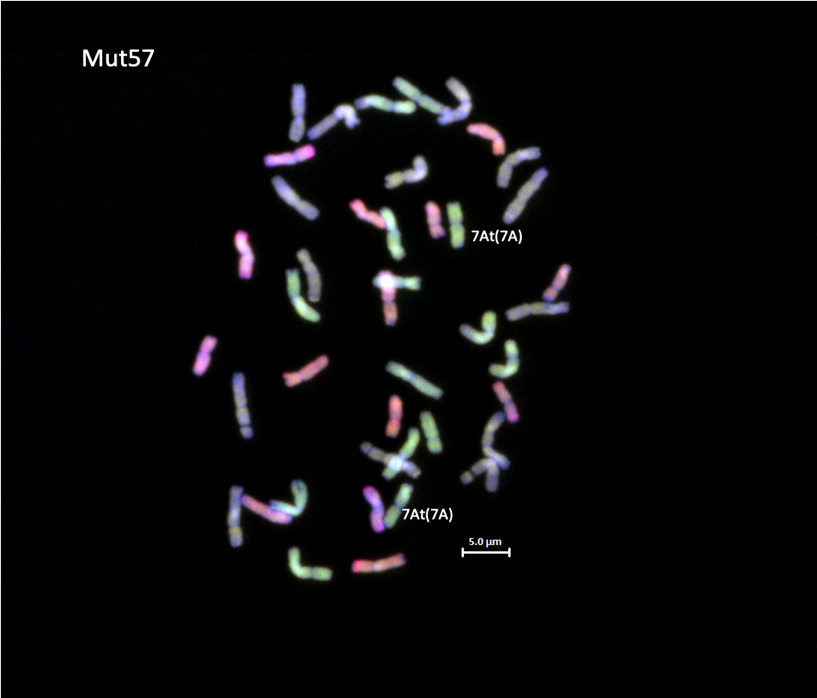

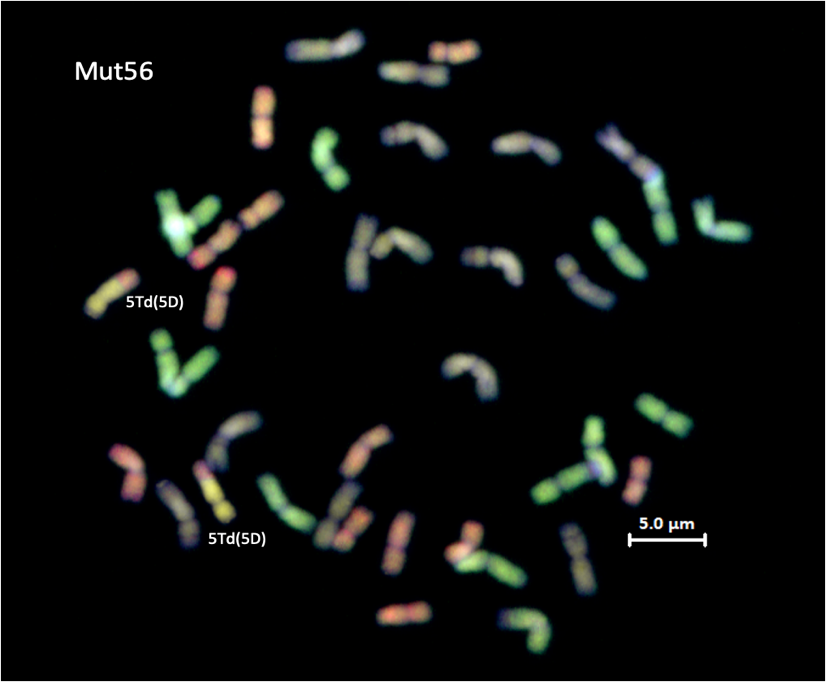

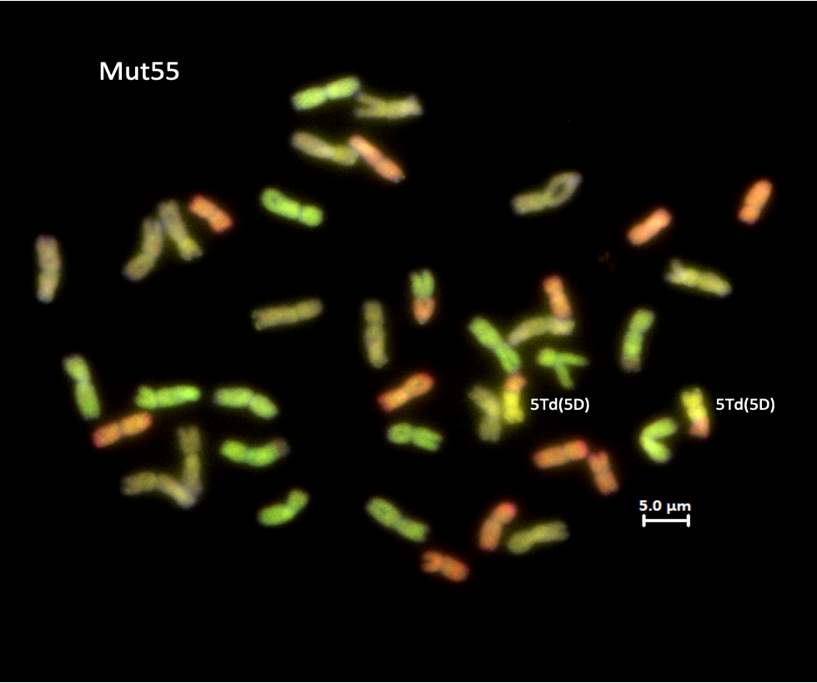

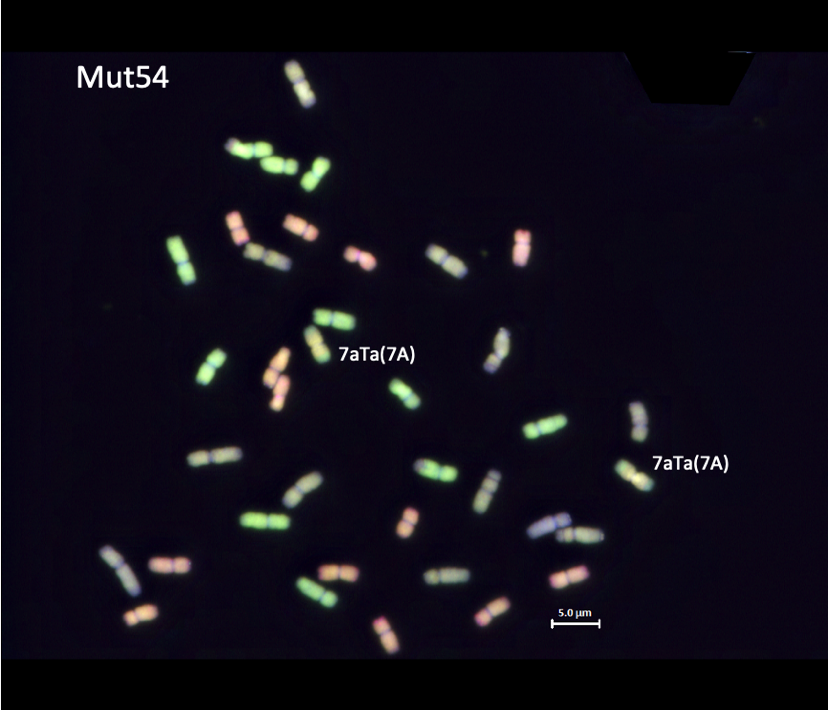

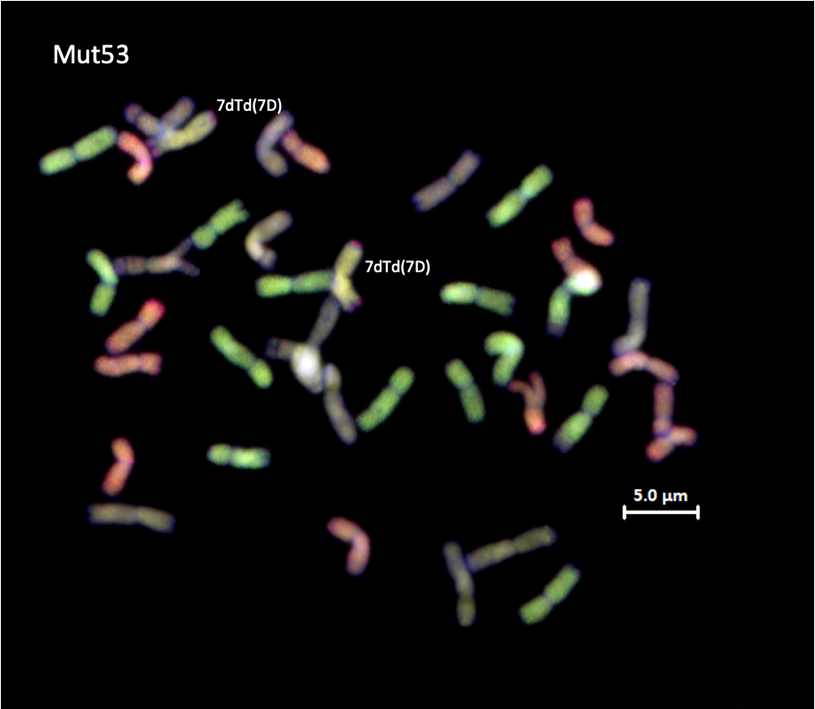


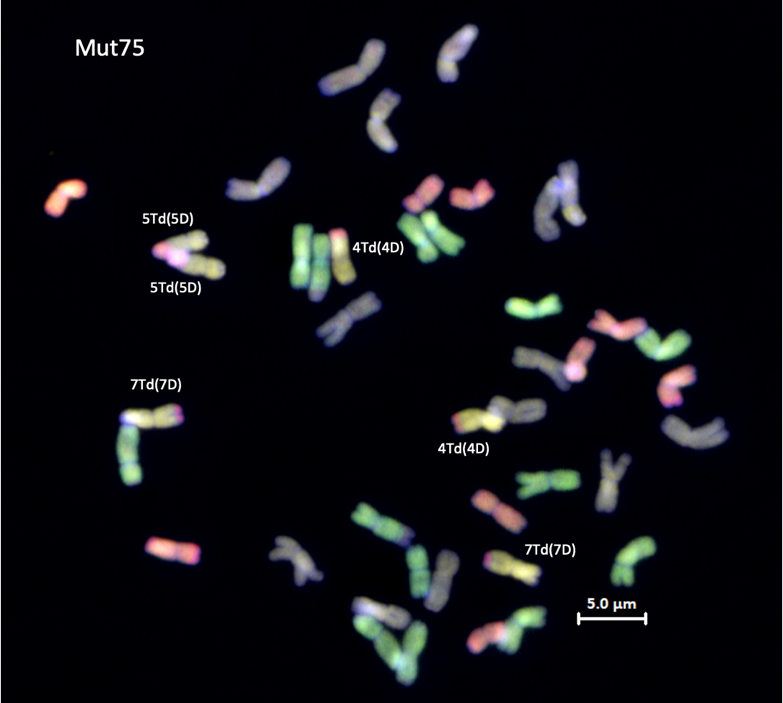

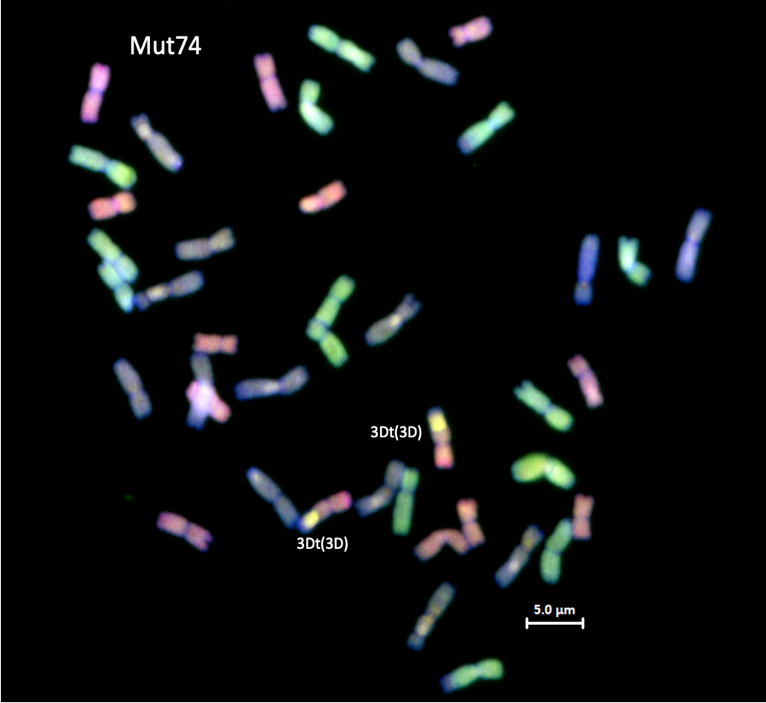

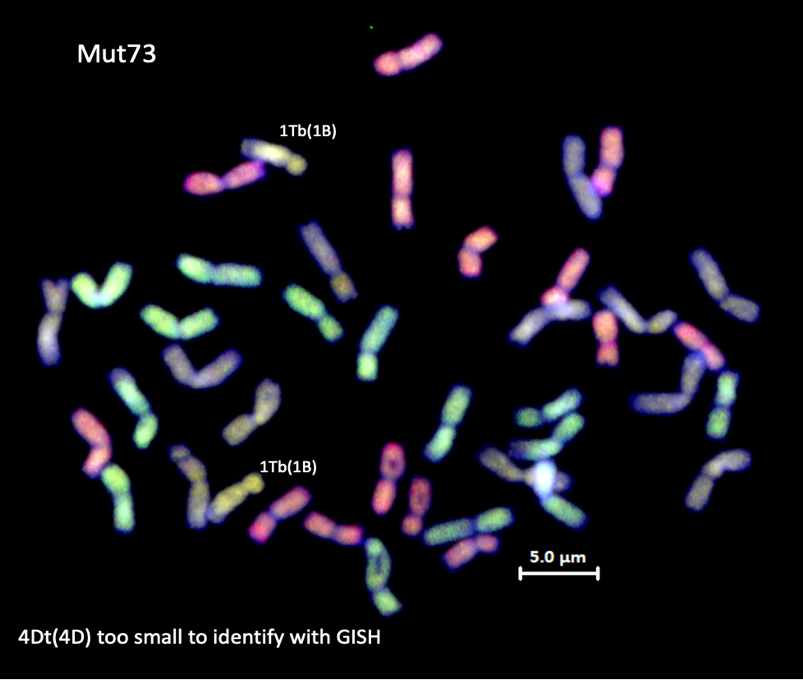

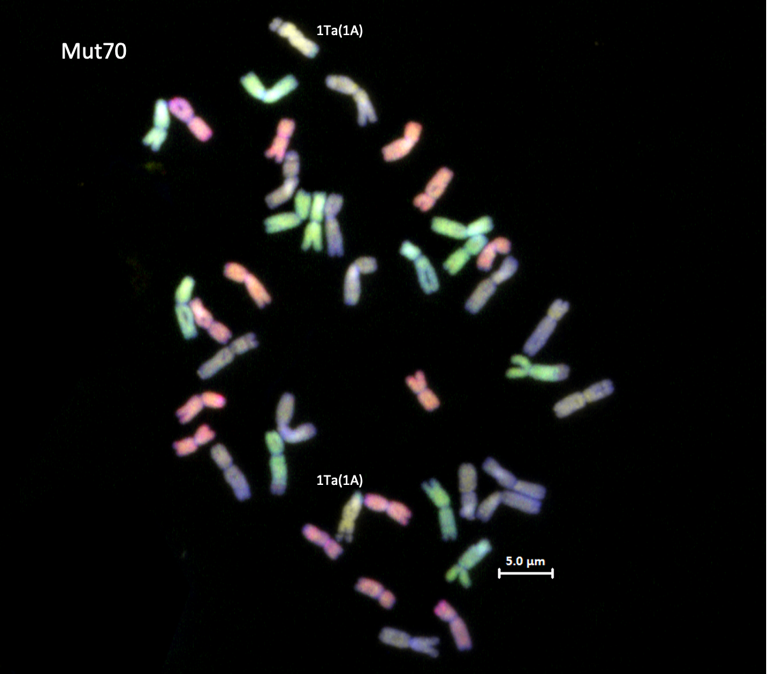

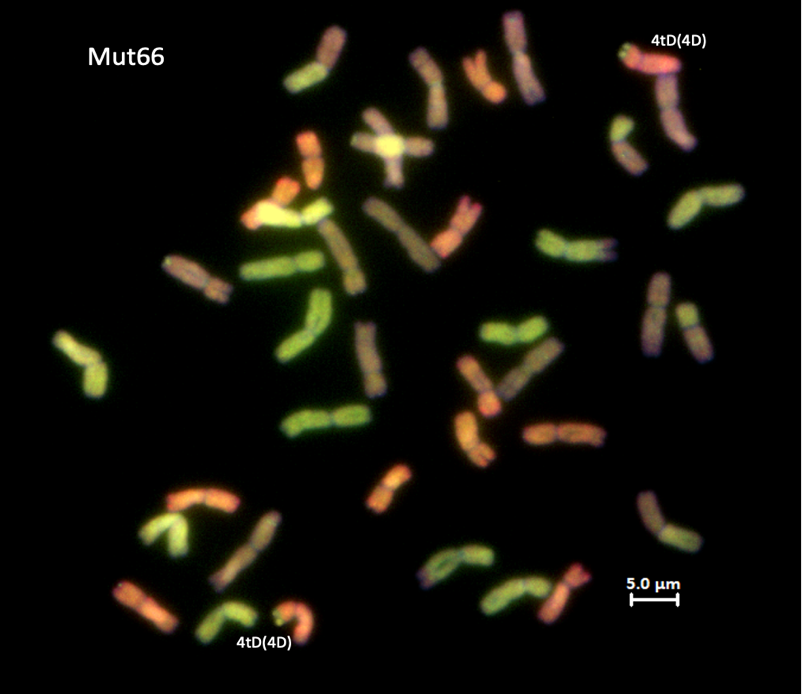

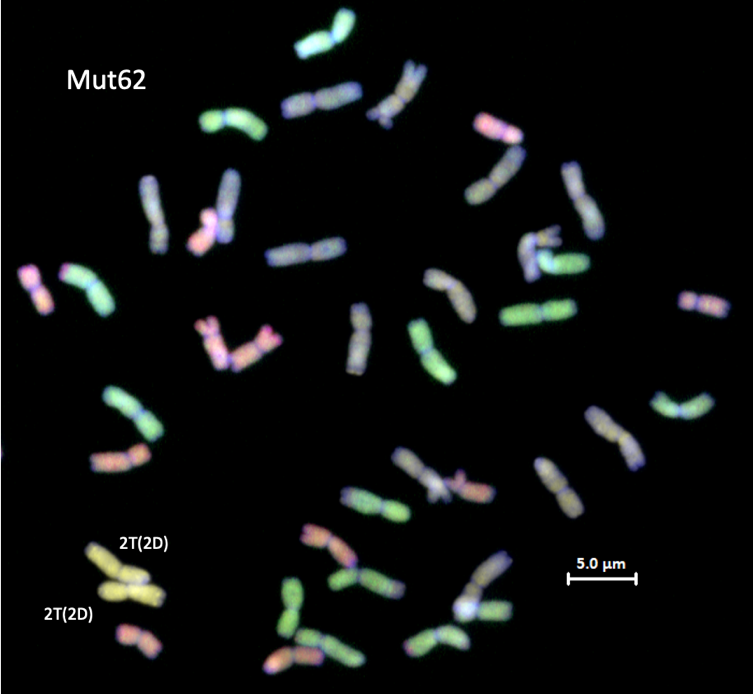


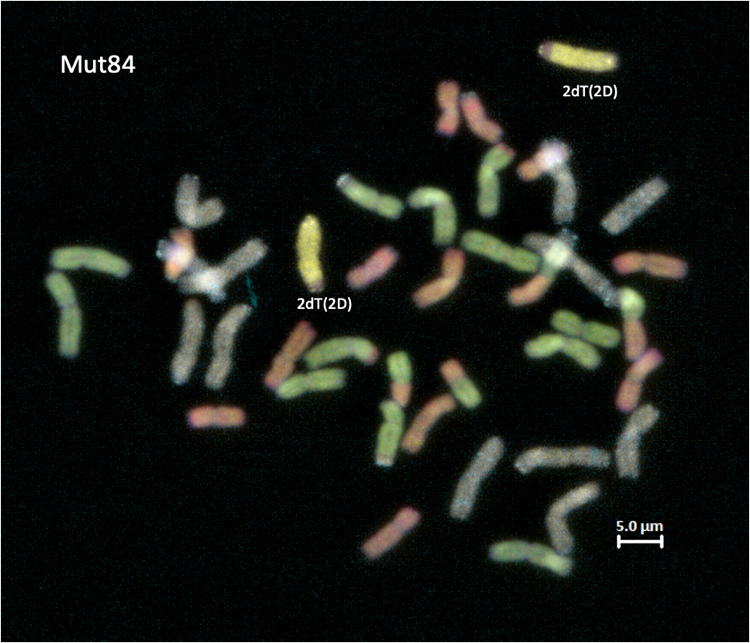

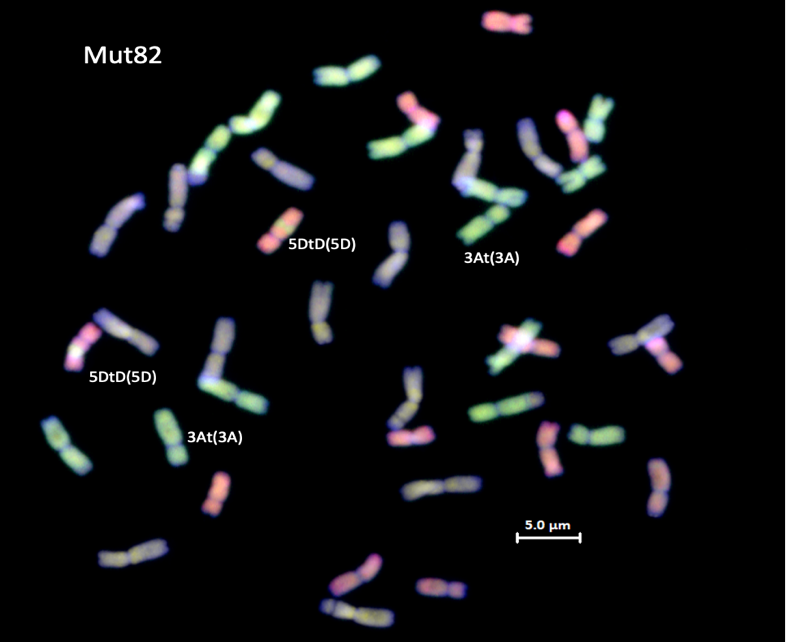

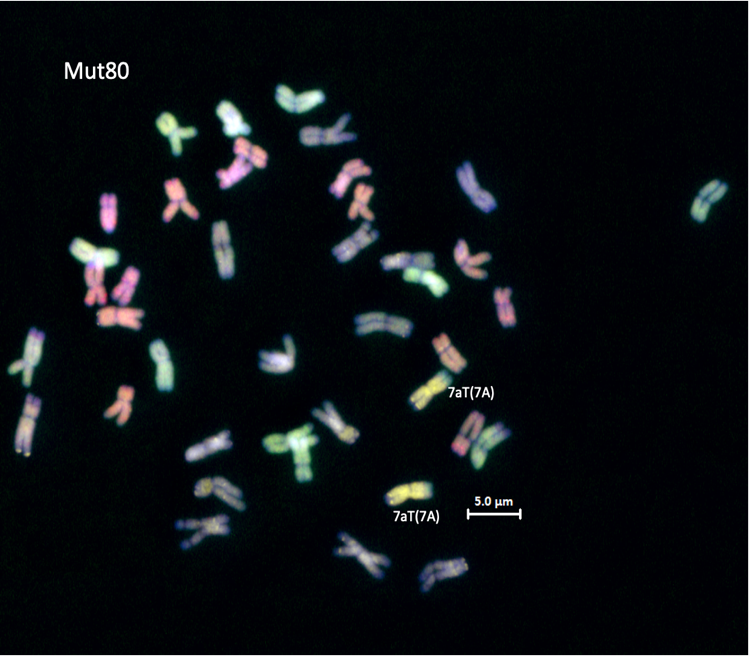

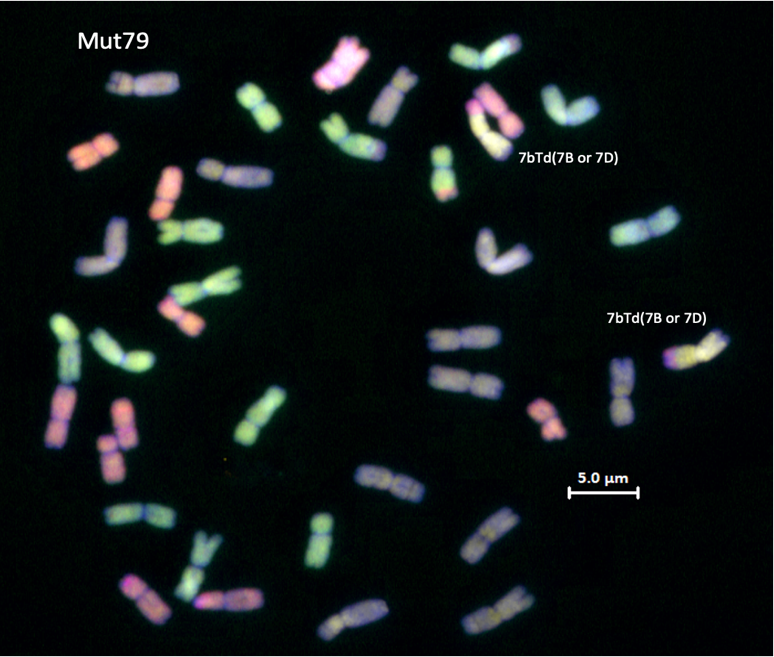

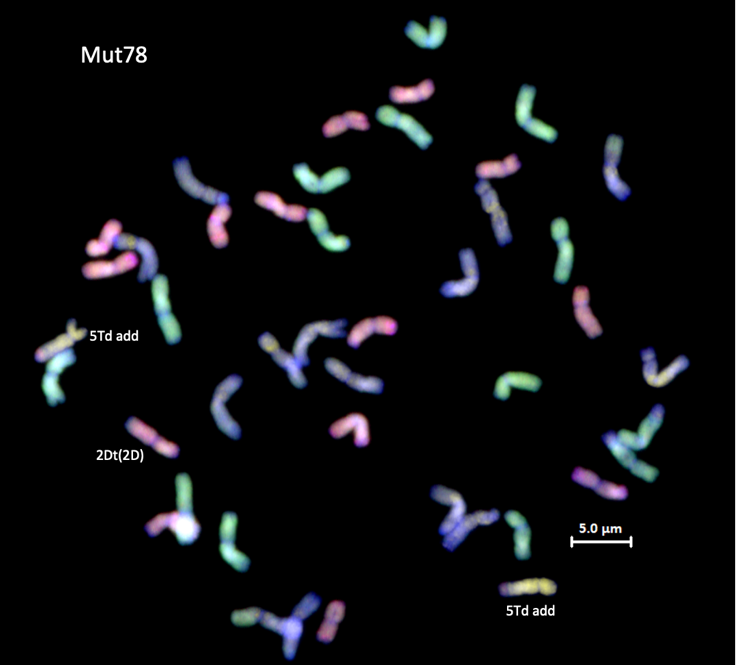

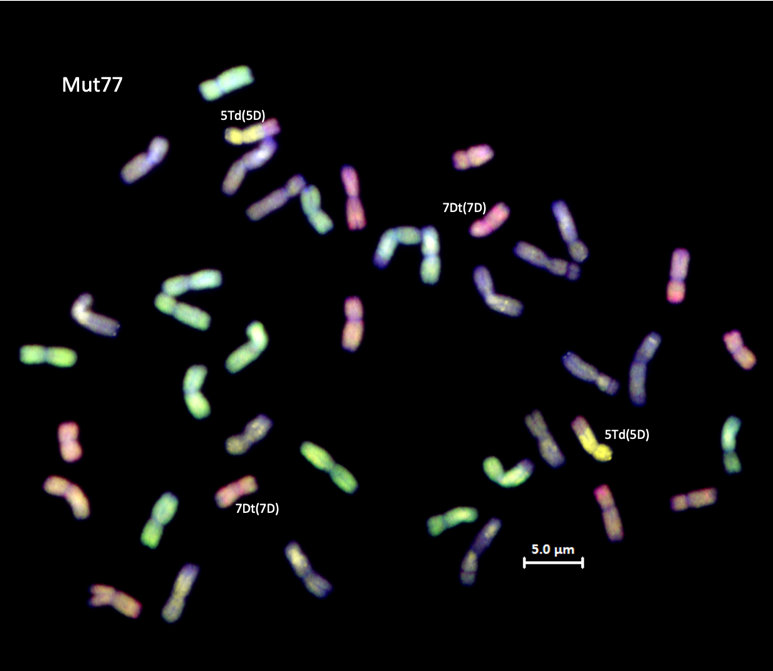


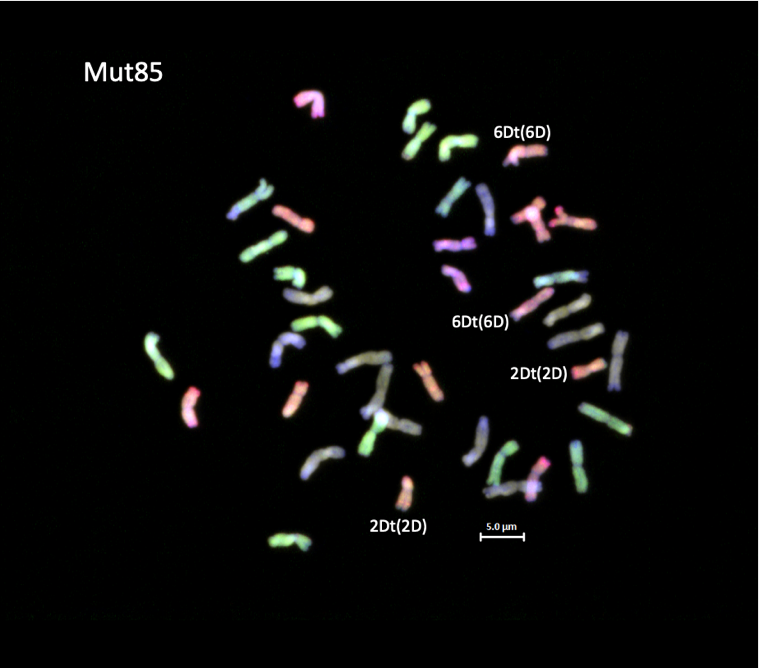

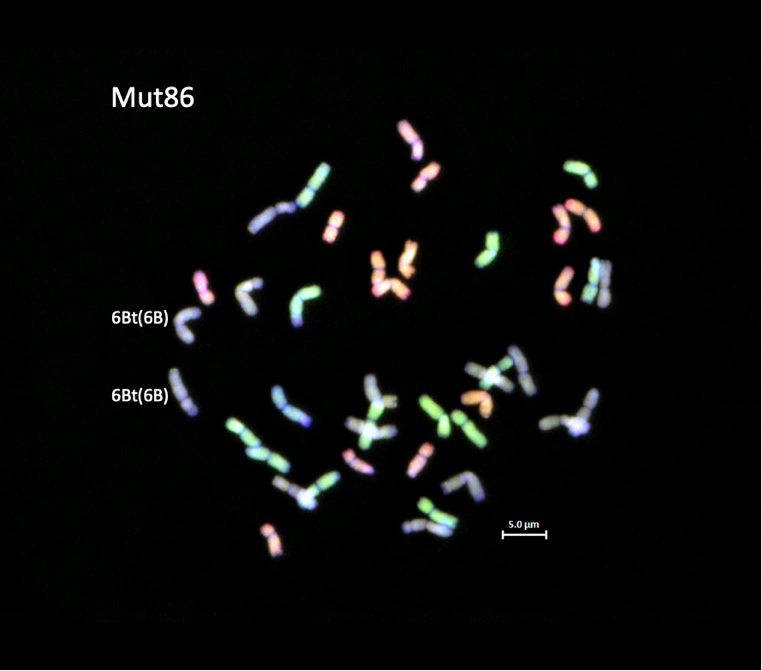

Supplement: Supplementary file 2 — Supplementary file2 (DOCX 29524 KB) [file 122_2026_5173_MOESM2_ESM.docx]
