# Supplementary figures and images for: The transfer of 98% of the genome of Aegilops mutica into wheat (Triticum aestivum)
Source: Theor Appl Genet. 2026 Feb 9;139(2):65. doi: 10.1007/s00122-026-05173-1 (PMC12886214; doi:10.1007/s00122-026-05173-1)

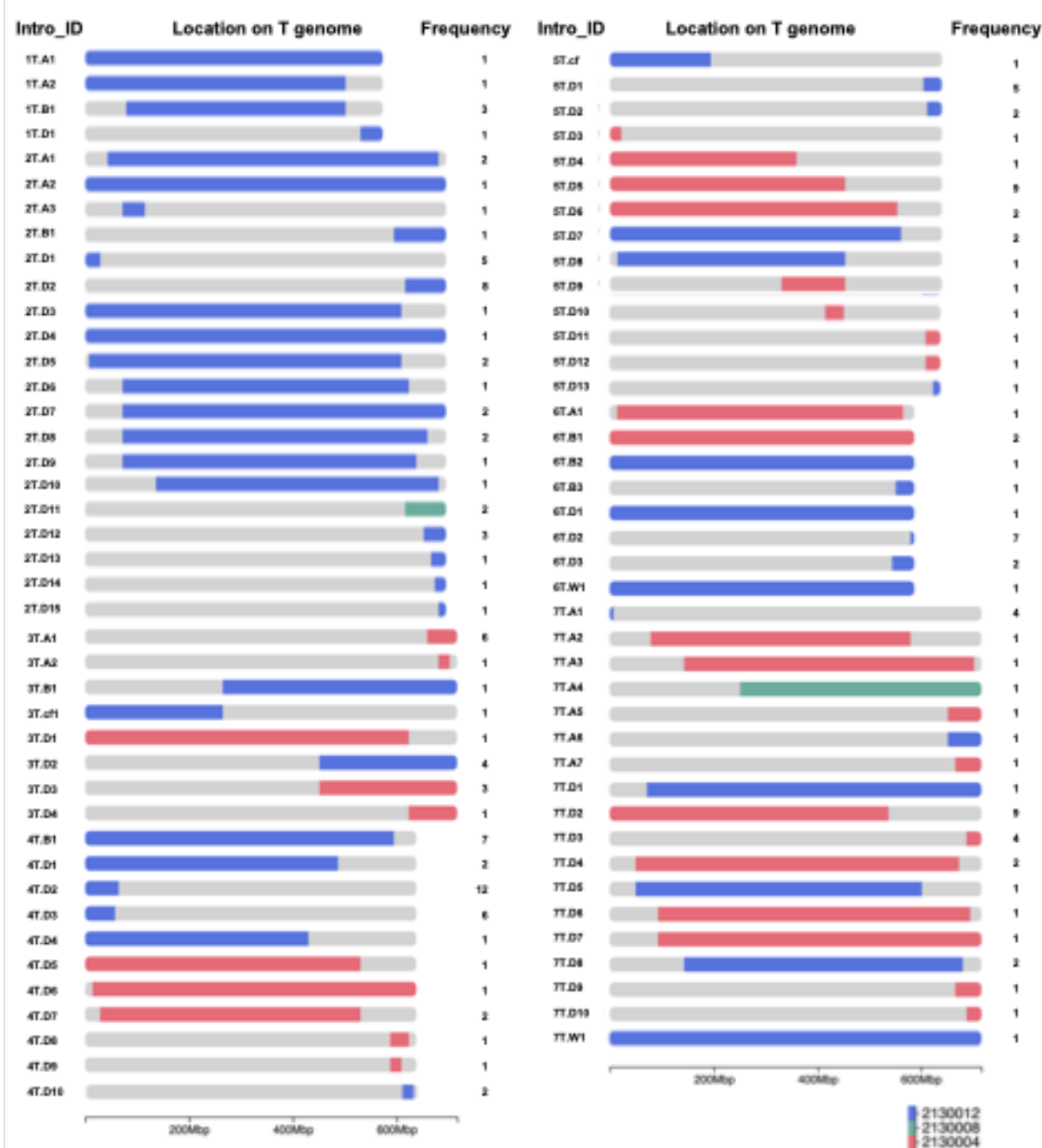

Supplement: Supplementary file 3 — Supplementary file3 (PDF 51 KB) [file 122_2026_5173_MOESM3_ESM.pdf]
